# Supplementary material for: Genome-wide analysis implicates inner ear development in Ménière disease
Source: Am J Hum Genet. 2026 Jun 16;113(7):1509–20. doi: 10.1016/j.ajhg.2026.05.011 (PMC13393102; doi:10.1016/j.ajhg.2026.05.011)
Supplement: Supplementary file 1 [file mmc1.pdf]

The American Journal of Human Genetics, Volume *113*

Supplemental information

Genome-wide analysis implicates inner ear development in Ménière disease

Zhuozheng Shi, Ravi Mandla, Jingjing Li, Xinzhe Li, Zixuan (Eleanor) Zhang, Sixing Chen, Sandra Lapinska, Alexander O. Flynn-Carroll, Bogdan Pasaniuc, Douglas J. Epstein, and Iain Mathieson

Supplemental Note

Power Analysis

To evaluate the ability of our study to detect genetic associations given the relatively low prevalence of Ménière's disease (MD) and case-control imbalance, we performed a statistical power analysis. Power was assessed as a function of minor allele frequency (MAF) and effect size (odds ratio, OR) at genome-wide significance ($P < 5\times10^{-8}$), using a standard non-centrality parameter framework for case-control association tests[1,2] (**Figure S1**). Specifically, for each combination of MAF and OR, we derived the expected allele frequency difference between cases and controls under a multiplicative model and computed the corresponding non-centrality parameter, accounting for the number of cases and controls in our study. Power was then obtained from the non-central chi-square distribution with one degree of freedom.

We observed that statistical power is primarily driven by the number of cases, given the extreme imbalance between cases and controls, with an effective sample size of 35,713. Under this framework, our study has high power (>80%) to detect common variants (MAF ≥ 0.05 and 0.1) with modest effect sizes (OR = 1.356 and 1.248), whereas substantially larger effects (OR = 1.929) are required for low-frequency variants (MAF = 0.01). These results indicate that our study is well-powered to identify common risk variants of modest effect but has limited sensitivity for detecting low-frequency variants unless their effects are large. This provides important context for interpreting both detected associations and the absence of signals at lower allele frequencies.

These power estimates should be interpreted in the context of the inherent challenges of studying a low-prevalence disorder such as Ménière's disease. With a population prevalence of approximately 0.2-0.5%, achieving a large case cohort requires aggregating data across multiple large-scale biobanks, as performed here. Our study, with 8,969 cases, represents one of the largest genetic studies of MD to date, and future progress will require additional MD-enriched cohorts.

References

1. Skol, A.D., Scott, L.J., Abecasis, G.R., and Boehnke, M. (2006). Joint analysis is more efficient than replication-based analysis for two-stage genome-wide association studies. Nat. Genet. *38*, 209–213. https://doi.org/10.1038/ng1706.

2. Purcell, S., Cherny, S.S., and Sham, P.C. (2003). Genetic Power Calculator: design of linkage and association genetic mapping studies of complex traits. Bioinformatics *19*, 149–150. https://doi.org/10.1093/bioinformatics/19.1.149.

Supplemental Figures

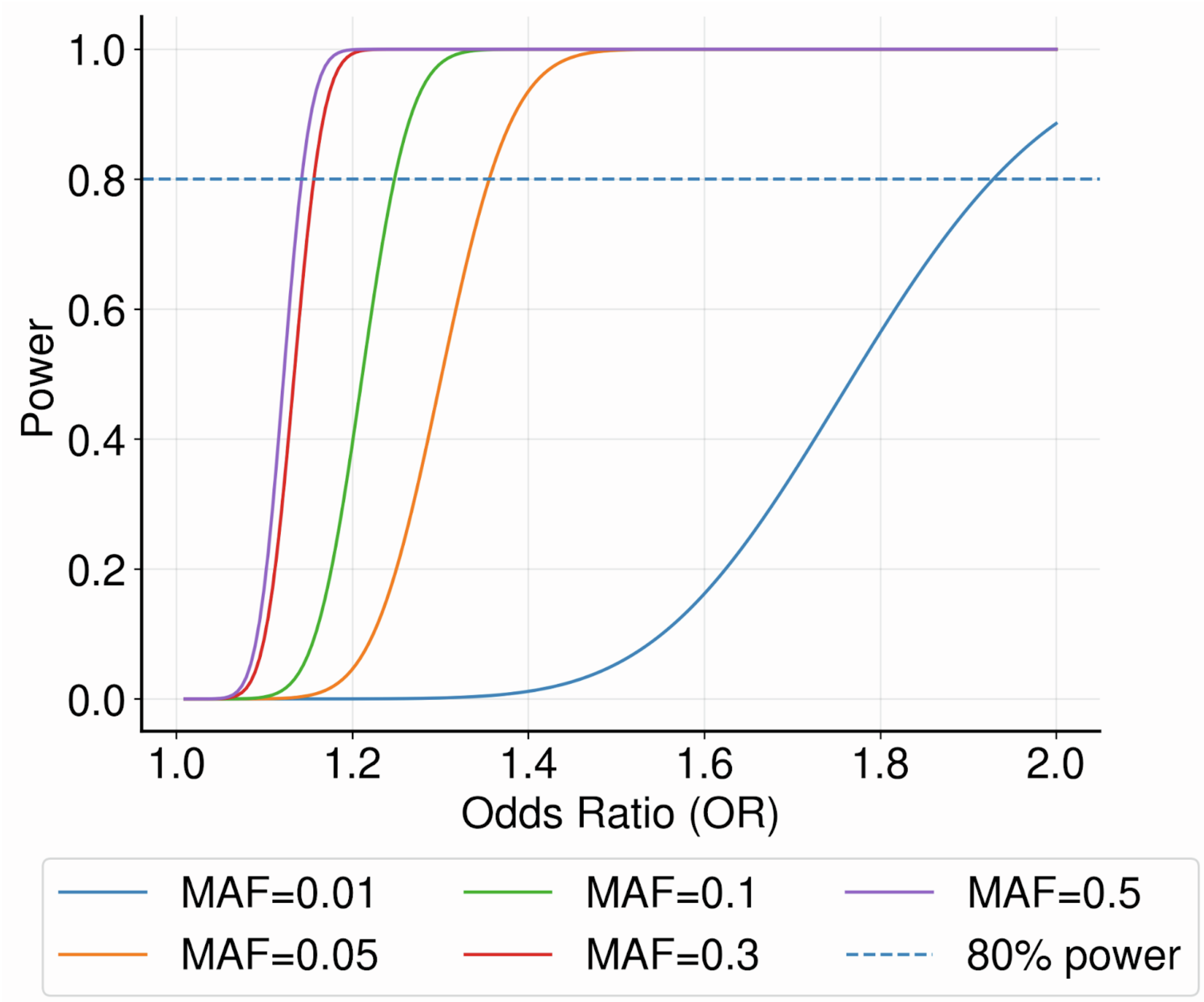

Figure S1. Power analysis for case–control GWAS.
Statistical power to detect genetic associations at genome-wide significance ($P < 5\times10^{-8}$) is shown as a function of odds ratio (OR) for variants with minor allele frequencies (MAF) of 0.01, 0.05, 0.1, 0.3, and 0.5 and 8,969 cases and 1,962,542 controls. The horizontal dashed line denotes 80% power.

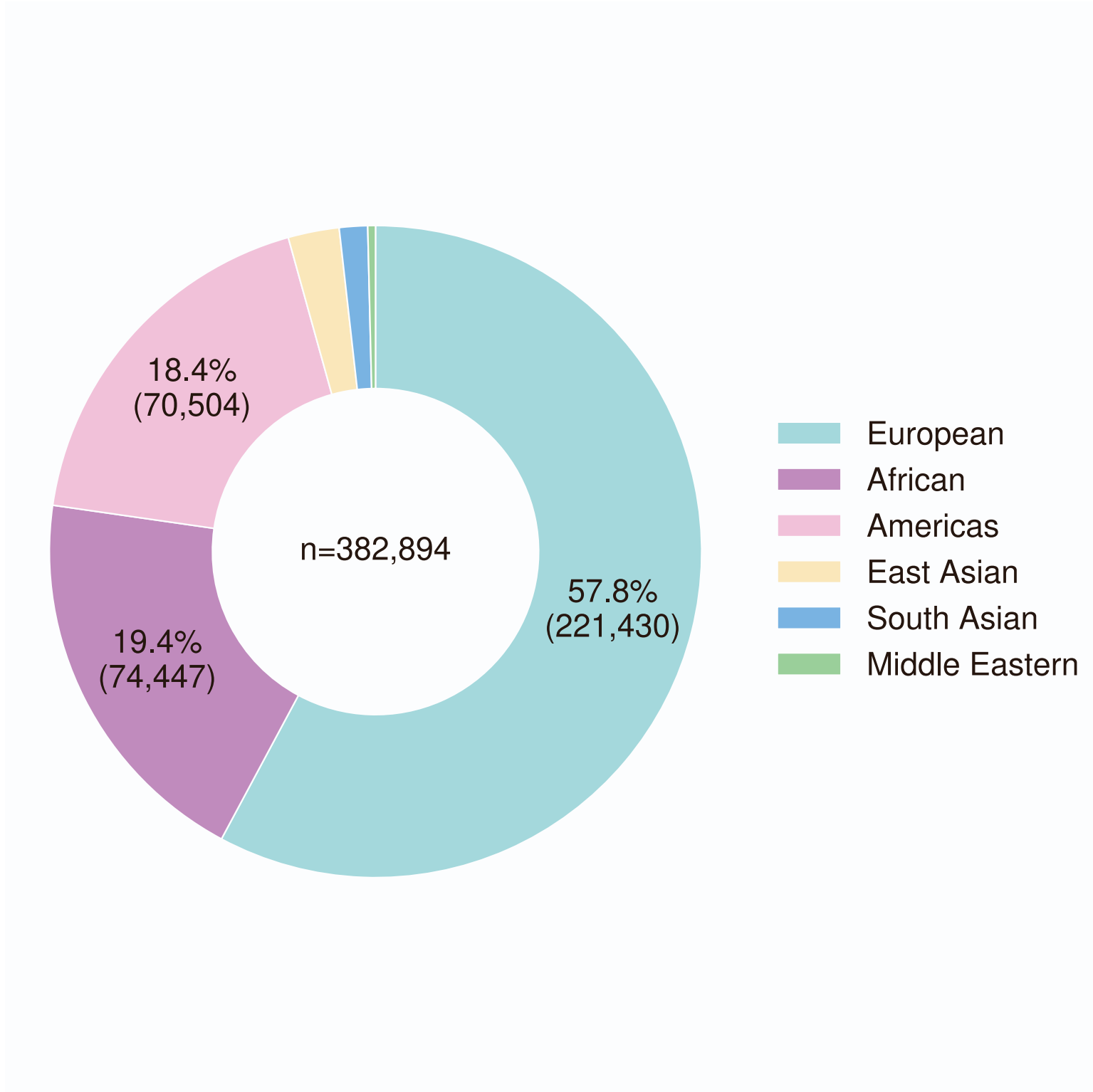

Figure S2. Genetically inferred ancestry distribution of all individuals in the All of Us version 8.

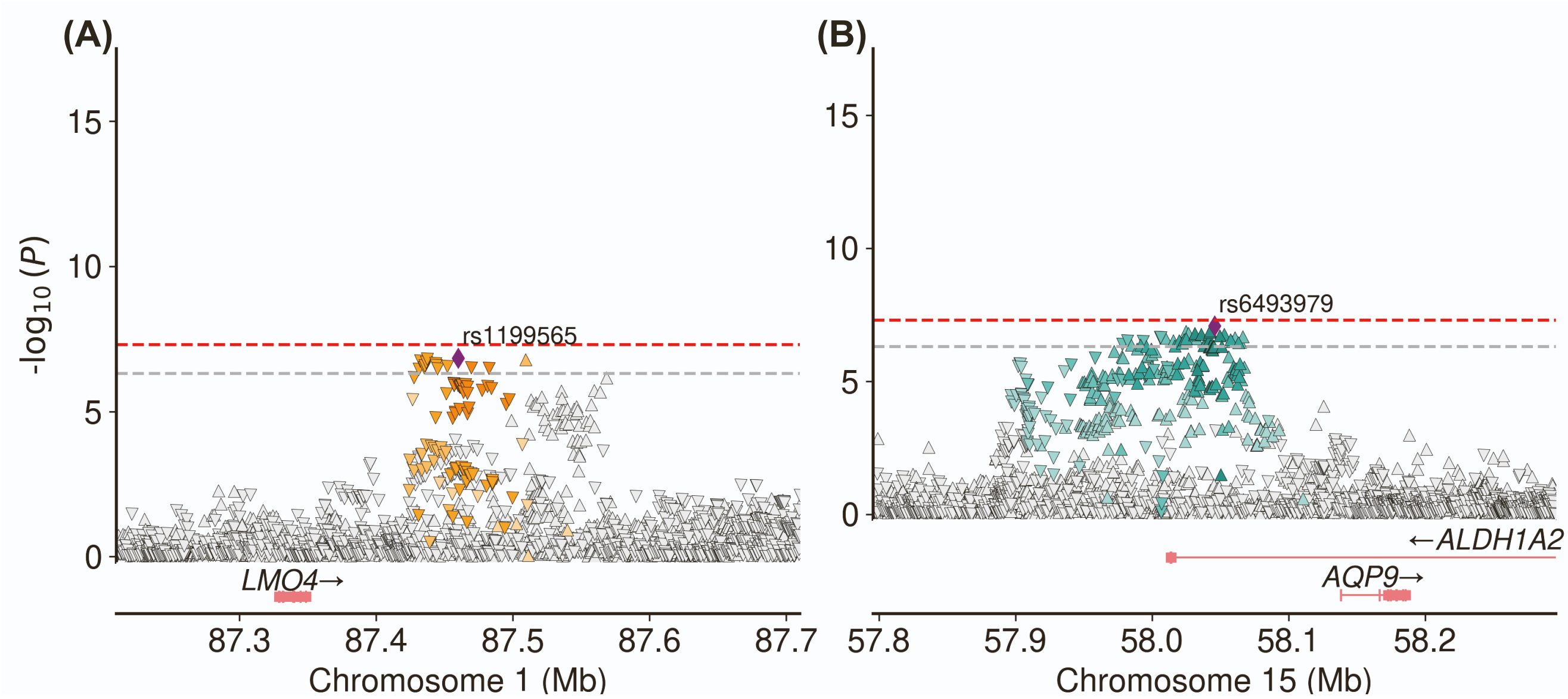

Figure S3. LocusZoom plot of suggestive loci for Ménière's disease.

Negative $\log_{10}$ P-values from the meta-analysis plotted against genomic positions for each index SNP region. The window size of the plots is 500kb. Genes of interest are colored in red. Red dotted line denotes genome-wide significance ($P<5\times10^{-8}$). Grey dotted line denotes suggestive significance ($5\times10^{-8}<P<5\times10^{-7}$).

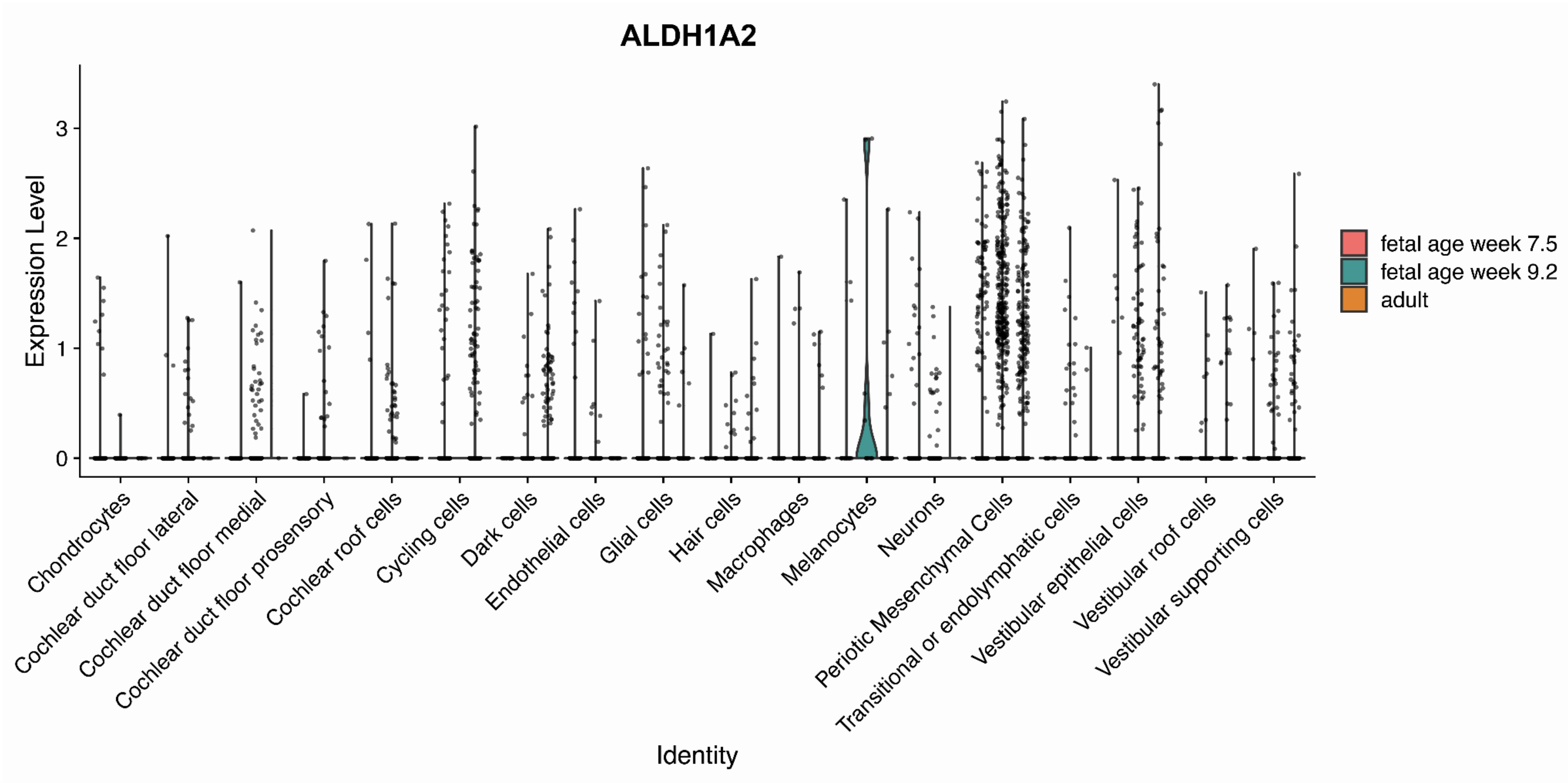

Figure S4. Violin plot of single-cell expression in ALDH1A2 from human inner ear scRNAseq across developmental stages.

A violin plot shows the expression of ALDH1A2 across diverse human inner ear cell types at three developmental stages (fetal week 7.5, fetal week 9.2, and adult). Data were obtained from the human inner ear atlas[60] *via the gEAR Dashboard*[61].

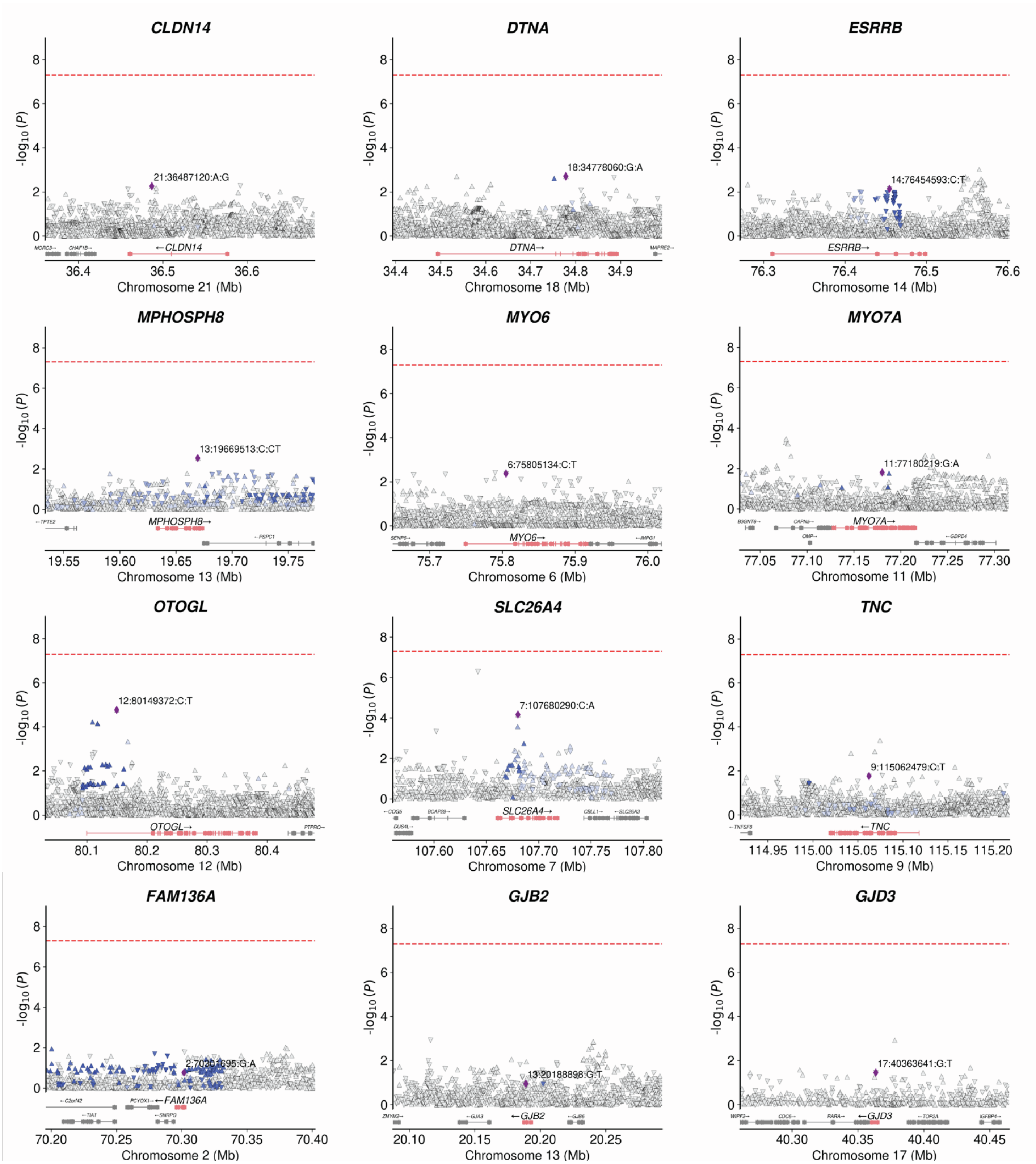
CLDN14
21:36487120:A:G
Chromosome 21 (Mb)
DTNA
18:34778060:G:A
Chromosome 18 (Mb)
ESRRB
14:76454593:C:T
Chromosome 14 (Mb)
MPHOSPH8
13:19669513:C:CT
Chromosome 13 (Mb)
MYO6
6:75805134:C:T
Chromosome 6 (Mb)
MYO7A
11:77180219:G:A
Chromosome 11 (Mb)
OTOGL
12:80149372:C:T
Chromosome 12 (Mb)
SLC26A4
7:107680290:C:A
Chromosome 7 (Mb)
TNC
9:115062479:C:T
Chromosome 9 (Mb)
FAM136A
Chromosome 2 (Mb)
GJB2
Chromosome 13 (Mb)
GJD3
17:40363641:G:T
Chromosome 17 (Mb)
-log10 (P)

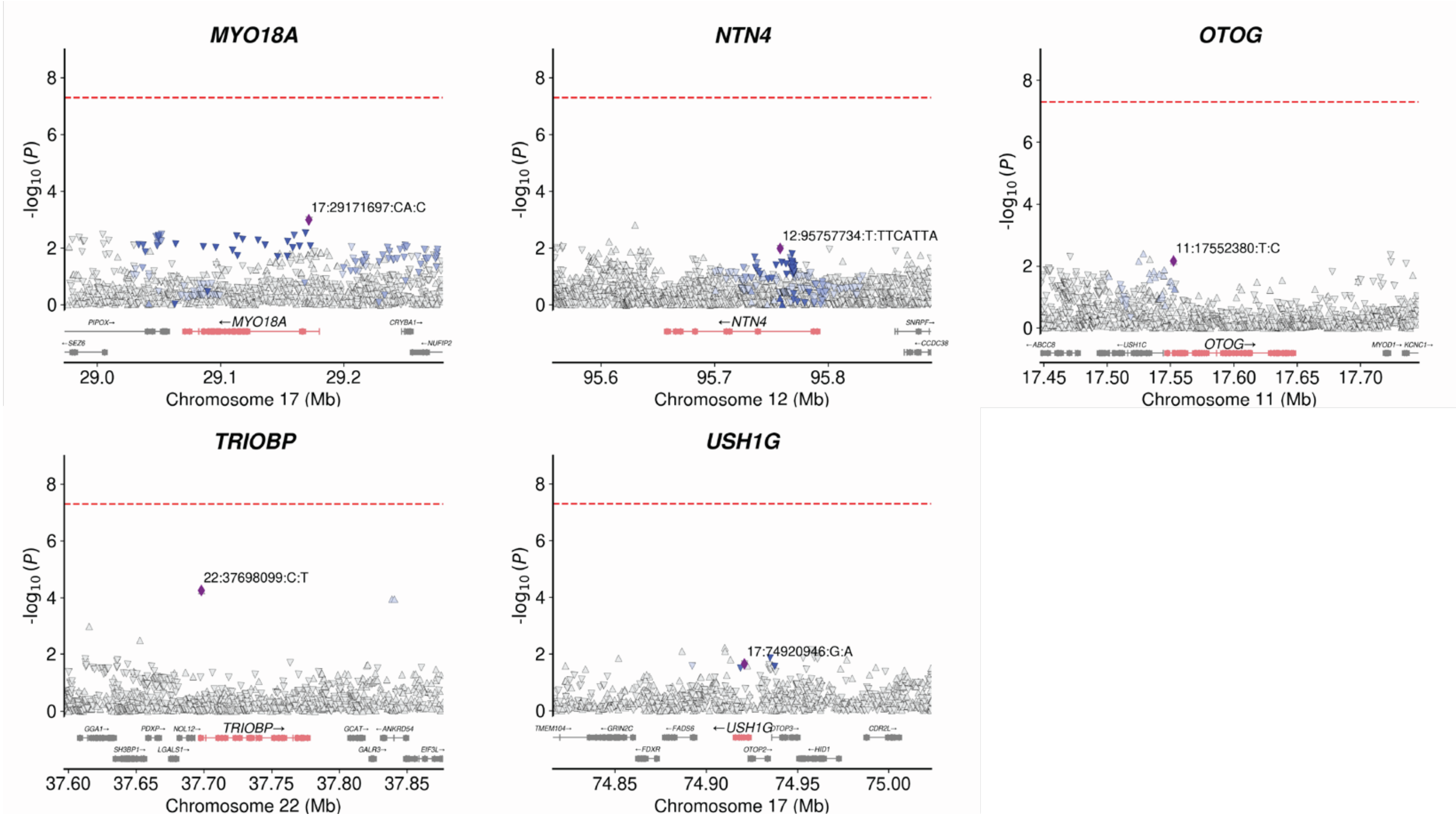

Figure S5: LocusZoom plots for regions around previously reported genes.

-log10 P-values from meta-analysis plotted against genomic positions for each previously reported gene[12-21]. Genes were identified via burden test or familial studies. The window size for the plots is ±100kb around the gene's start and end positions. Genes of interest were colored in red along the x-axis. Red dotted line denotes genome-wide significance (P<5×10-8). The variant with the lowest P-value and non-zero MAF in the 1000 Genomes EUR within each gene body was annotated.
