# Supplementary material for: Genome-wide analysis implicates inner ear development in Ménière disease
Source: Am J Hum Genet. 2026 Jun 16;113(7):1509–20. doi: 10.1016/j.ajhg.2026.05.011 (PMC13393102; doi:10.1016/j.ajhg.2026.05.011)
Supplement: Supplementary file 2 [file mmc3.pdf]

Genome-wide analysis implicates inner ear development in Ménière disease

Authors

Zhuozheng Shi, Ravi Mandla, Jingjing Li, ..., Bogdan Pasaniuc, Douglas J. Epstein, Iain Mathieson

Correspondence

zhuozheng.shi@pennmedicine.upenn.edu (Z.S.), mathi@pennmedicine.upenn.edu (I.M.)

A genome-wide association study of Ménière disease across five biobanks identifies genome-wide significant associations at *EYA4*, *EYA1*, and *CYP26A1*. These genes regulate inner ear development and retinoic acid signaling, establishing a genetic framework linking developmental pathways to this vestibular disorder.

Shi et al., 2026, The American Journal of Human Genetics *113*, 1509–1520
July 2, 2026 © 2026 The Authors. Published by Elsevier Inc. on behalf of American Society of Human Genetics.
https://doi.org/10.1016/j.ajhg.2026.05.011

CellPress

Genome-wide analysis implicates inner ear development in Ménière disease

Zhuozheng Shi,[1,2,*] Ravi Mandla,[1,2] Jingjing Li,[1] Xinzhe Li,[1,2] Zixuan (Eleanor) Zhang,[1] Sixing Chen,[1] Sandra Lapinska,[1,2] Alexander O. Flynn-Carroll,[1,2] Bogdan Pasaniuc,[1,3,4,5] Douglas J. Epstein,[1,5] and Iain Mathieson[1,5,*]

Summary

Ménière disease (MD) is a chronic inner ear disorder characterized by recurrent vertigo, fluctuating sensorineural hearing loss, and tinnitus, but its etiology remains poorly understood. We performed a genome-wide meta-analysis of 8,969 MD case subjects and 1,962,542 control subjects across five biobanks, identifying five independent genome-wide significant loci and estimating an observed-scale SNP heritability of 7% (SE 0.8%), indicating a modest but significant contribution of common genetic variation to MD risk. The genome-wide significant signals comprise two independent variants at *EYA4*, two at *EYA1*, and one near *CYP26A1*, with odds ratios between 1.1 and 1.25. Associations at two additional loci, *LMO4* and *ALDH1A2*, fell just below the genome-wide significance threshold ($5 \times 10^{-8} < p < 5 \times 10^{-7}$). Fine-mapping and integrative functional analyses implicate two convergent biological processes: developmental regulation of the inner ear, involving *EYA4*, *EYA1*, and *LMO4*, and retinoic acid metabolism, with associations near *CYP26A1/C1* and *ALDH1A2*, suggesting disrupted retinoic acid signaling in sensory and fluid-pressure homeostasis. These developmental regulator genes are robustly expressed in fetal and adult human inner ear cell types, supporting a model in which altered developmental programs predispose individuals to adult vestibular and auditory dysfunction. Phenome-wide and genetic correlation analyses further reveal shared genetic architecture between MD and related traits, including vertigo, tinnitus, hearing loss, migraine, and sleep apnea, situating MD within a broader spectrum of sensory and neurological disorders. Collectively, these findings establish a genetic framework for Ménière disease risk and implicate developmental regulators of the inner ear and retinoic acid signaling as key contributing pathways.

Introduction

Ménière disease (MD) is a chronic inner ear disorder characterized by debilitating episodes of vertigo, fluctuating sensorineural hearing loss (SNHL), tinnitus, and aural fullness.[1–3] The etiology of MD remains unclear, but it is frequently associated with endolymphatic hydrops, an abnormal accumulation of endolymph within the inner ear.[4] Several pathophysiological mechanisms have been proposed, including fluid imbalance, infection, and vascular dysfunction, but these hypotheses lack direct genetic or molecular support.[5]

MD affects approximately 1 in 2,000 individuals, typically presenting in middle to late adulthood, with higher prevalence among women and those of European ancestry.[6–10] Genetic studies indicate a heritable contribution to MD risk, with familial cases (5%–15% of affected individuals) following autosomal-dominant inheritance with ~60% penetrance.[11] Rare coding variants in genes such as *OTOG*, *GJD3*, *FAM136A*, and *DTNA* have been identified in familial MD.[12–21] However, the genetic basis of sporadic MD, which constitutes the majority of cases, remains poorly understood.[5] The contribution of common variants and the proportion of risk they explain have not been systematically defined, leaving the full genetic architecture and downstream molecular mechanisms unresolved.

Recently, large-scale initiatives to expand biobank sample sizes, harmonize phenotypic data, and perform high-quality genome-wide genotyping across diverse populations have transformed the study of complex traits at scale.[22–28] These resources enable well-powered genome-wide association studies (GWASs) and meta-analyses, even for low-prevalence disorders.[29–31] Furthermore, meta-analyses across biobanks allow for detection of novel risk loci, refinement of heritability estimates, and mapping of genetic variants to molecular function, thereby providing opportunities to address critical gaps in our understanding of the genetic architecture of MD.

We leveraged genome-wide summary statistics from five major biobanks—UK Biobank (UKB), All of Us (AoU), Million Veteran Program (MVP), FinnGen, and Biobank Japan (BBJ)—to characterize the genetic architecture of MD. Through meta-analysis of 8,969 MD case subjects and 1,962,542 control subjects from three populations across these biobanks, and downstream fine-mapping and integrative annotation, we identified five independent genome-wide significant signals at three loci and an SNP-based heritability of 7%. Four of these signals

[1]Department of Genetics, Perelman School of Medicine, University of Pennsylvania, Philadelphia, PA, USA; [2]Graduate Group in Genomics and Computational Biology, Perelman School of Medicine, University of Pennsylvania, Philadelphia, PA, USA; [3]Department of Biostatistics, Epidemiology and Informatics, Perelman School of Medicine, University of Pennsylvania, Philadelphia, PA, USA; [4]Department of Computer and Information Science, School of Engineering and Applied Sciences, University of Pennsylvania, Philadelphia, PA, USA
[5]These authors contributed equally
*Correspondence: zhuozheng.shi@pennmedicine.upenn.edu (Z.S.), mathi@pennmedicine.upenn.edu (I.M.)
https://doi.org/10.1016/j.ajhg.2026.05.011.
© 2026 The Authors. Published by Elsevier Inc. on behalf of American Society of Human Genetics.
This is an open access article under the CC BY license (http://creativecommons.org/licenses/by/4.0/).

map to potential regulatory elements for *EYA4* and *EYA1*, genes essential for inner ear development. These associations, combined with suggestive evidence at *LMO4*, highlight a developmental regulatory mechanism. Additional signals near *CYP26A1/C1* and *ALDH1A2* implicate retinoic acid signaling, a pathway critical for sensory and fluid-pressure homeostasis. Phenome-wide and genome-wide correlation analyses further demonstrate shared genetic architecture between MD and non-symptom traits, including migraine ($r_g = 0.28$) and sleep apnea ($r_g = 0.20$). Collectively, these findings support a polygenic basis for MD driven by regulatory variation in developmental and morphogen pathways, providing a genetic framework for understanding its complex etiology.

Subjects and methods

Contributing biobanks and GWASs

AoU Research Program

AoU is a US-based biobank with genomics data, electronic health records (EHRs), and survey information from diverse populations.[24] We performed a GWAS of MD using Regenie,[31] including age, sex, age^2, age^2 × sex, and the top 10 principal components (PCs) as covariates. We obtained genotypes from short-read whole-genome sequencing in the Curated Data Repository (CDR) v.8 release and defined phenotypes using phecode 386.1. We filtered variants by population-specific allele frequency >1% or allele count >100 in any ancestry subgroup. We performed additional quality control in PLINK 2 using –maf 0.01, –geno 0.02, and –mind 0.05 across all individuals. Related individuals were removed. We defined case subjects as participants with ≥2 phecode encounters and controls as those with none.

MVP

MVP is a large and ancestrally diverse US biobank linking genomic data to detailed EHR and survey information.[32] We used summary statistics provided by MVP from the genetically inferred ancestry (GIA) gwPheWAS release,[27] where the MD GWAS (phecode 386.1) was performed with SAIGE,[33] among European and Admixed American participants (2,306 case subjects, 507,752 control subjects). Case subjects were defined as participants with ≥2 phecode encounters and controls as those with none. Ancestry was inferred via principal-component analysis (PCA)-based random forest classifiers. Genotypes were imputed using a hybrid reference panel of African Genome Resources + 1000 Genomes Phase 3 v.5 and filtered by imputation quality >0.3, minor-allele count >20, call rate >0.975 for minor-allele frequency (MAF) >0.01, and >0.99 for MAF <0.01.[27]

UKB

The UKB is a prospective cohort of ∼500,000 participants with genotype and EHR data from the United Kingdom.[23] We used the Pan-UKB European-ancestry GWAS summary statistics (phenocode 20002, coding 1421, SAIGE model),[25,33] which included 1,220 case subjects and 419,253 control subjects. Genetic ancestry was inferred using PCA-based random forest classifiers, and only unrelated individuals (PC-Relate) were included. Genotypes were imputed to the UKB version 3 panel (∼97 million variants) and filtered by INFO >0.8 and minor-allele count >20.[25]

FinnGen

FinnGen is a nationwide research initiative combining Finnish biobank data with health registry records.[26] We used GWAS summary statistics from the R12 release for endpoint H8_Ménière, analyzed with Regenie. The study included 3,312 case subjects and 474,839 control subjects of European ancestry. Phenotypes were harmonized across International Classification of Diseases (ICD)-10 (H810), ICD-9 (3860), and ICD-8 (38599) codes. Genotypes were imputed to the population-specific SISu v.4.2 reference panel and filtered by INFO >0.9 and MAF >0.01.

BBJ

BBJ is a large hospital-based biobank that links genomic data with clinical information from ∼200,000 Japanese participants across phenotypes.[28,34] The BBJ GWAS was conducted using SAIGE to test for associations between ∼13 million imputed variants (imputed to the 1000 Genomes Phase 3 East Asian panel) and clinically ascertained diagnoses. For MD, the analysis included 1,290 case subjects and 177,436 control subjects, defined by phecode 386.1. Quality control followed the BBJ protocol[34]: variants with MAF <0.01, imputation INFO <0.8, or Hardy-Weinberg $p < 1 \times 10^{-6}$ were excluded, and participants with call rate <0.98 or excess heterozygosity were removed. Association testing by Sakaue et al.[34] adjusted for age, sex, top 20 genetic PCs, and genotyping batch. Our analysis is based on these summary statistics.

Ancestry definition

Genetic ancestry assignments for AoU, the MVP, and UKB were obtained from each cohort, where ancestry inference was performed using PCA and random forest classifiers trained on the Human Genome Diversity Project and the 1000 Genomes Project.[24,25,27,35,36] In FinnGen and BBJ, participants were recruited from genetically homogeneous Finnish and Japanese populations, corresponding broadly to European and East Asian ancestries, respectively.

GWAS meta-analysis

We conducted the meta-analysis using METAL with summary statistics from the five GWASs described above.[24–27,34,37] We lifted over Pan-UKB and BBJ summary statistics from GRCh37 to GRCh38 using the UCSC liftOver tool.[38] We kept single study-private variants and removed variants with MAF <0.01 or with minor-allele count <10. We applied genomic control to all datasets before meta-analysis and again on the meta-analyzed result. We used the STDERR scheme to perform meta-analysis, utilizing weighted effect size estimates and inverted standard errors. We calculated the effective sample size to replace the overall sample size.[37] Cochran's Q and its p value as measures of heterogeneity were calculated by METAL. We had 14,096,168 SNPs after meta-analysis and kept a total of 13,423,260 that are also in the 1000 Genomes high-coverage Phase 3 reference panel[35] for downstream analyses.

Heritability estimate

We estimated SNP-based heritability using LD Score Regression[39] (LDSC). Meta-analysis summary statistics for MD were first processed using the munge_sumstats.py script provided with LDSC. We excluded strand-ambiguous SNPs and aligned all variants to the 1000 Genome Phase 3 high-coverage reference genome.[35] For linkage disequilibrium (LD) reference, we computed LD scores using 632 European populations from the 1000 Genomes Project Phase 3 high-coverage data.

We estimated heritability on the observed scale, assuming a case-control design with 8,969 MD case subjects and 1,962,542 control subjects. We also attempted, but failed, to convert

observed-scale heritability to liability-scale heritability using an assumed population prevalence of 0.2% and the methods of Lee et al. and Ojavee et al.[40,41]

Index SNP selection

We defined Genome-wide significant hits at $p < 5 \times 10^{-8}$ and suggestive associations at $p < 5 \times 10^{-7}$. We obtained index SNPs, or index hits, through LD clumping using Plink 1.9, with –clump-r2 of 0.05 and –clump-kb of 1 million bp.[42] Genotype data of 632 European individuals from the 1000 Genomes Project Phase 3 high-coverage variant data were used as reference panels.[35] The clumping threshold for p value is first set to 5×10^{-8} and then loosened to 5×10^{-7} for regions previously having no clumped result. Loci with p values below the thresholds but clumped with no other variants were defined as significant or suggestive loci without support. We included individual summary statistics for each significant and suggestive locus in Table S22 for each cohort and the meta-analysis.

Variant consequences

To predict the consequences of the variants, we employed Ensembl Variant Effect Predictor (VEP).[43] The input to VEP was the list of SNPs that are significant and clumped with each index SNP.

Fine-mapping

To localize putative causal variants within genome-wide significant regions, we performed statistical fine-mapping using the sum of single effects (SuSiE).[44] The fine-mapping window was ± 500 kb centered on each index SNP and the midpoint of two index SNPs on chromosome 6. For each region, SNPs that were present across the five contributing biobanks were kept. We constructed an ancestry-weighted LD panel by effective sample size in the meta-analyzed GWAS from the 1000 Genomes Project Phase 3 high-coverage data.[35] We confirmed accurate LD matching between the reference and meta-analysis data by assessing LD–Z concordance ($s \leq 0.003$ across all loci). For each locus, SuSiE was run with up to 10 potential causal components ($L = 10$) and default prior variance settings. 95% credible sets (CSs) were defined as the minimal subset of variants whose cumulative posterior inclusion probabilities (PIP) reached 0.95.

PheWAS of index SNPs

To assess the phenotypic pleiotropy of lead SNPs from the MD GWAS meta-analysis, we queried publicly available PheWAS results from the FinnGen-MVP-UKB meta-analysis, accessed via FinnGen Data Freeze 12.[26] For each index SNP, we extracted its association statistics across a curated panel of 330 harmonized phenotypes. Only associations reaching Bonferroni significance of 1.5×10^{-4} were considered statistically significant.

eQTL characterization

To assess regulatory relevance, we checked whether each index SNP was a significant *cis*-eQTL in any tissue based on GTEx v.10 single-tissue *cis*-eQTL summary statistics.[45] We considered variant-gene pairs passing permutation-based significance thresholds (false discovery rate [FDR] < 0.05) across 50 tissues.

MAGMA gene association

We performed gene-level association analysis using MAGMA to map SNP-level GWAS meta-analysis summary statistics to gene-level signals, where individual SNP p values were combined into gene test statistics.[46] We mapped SNPs in meta-analysis summary statistics to genes through annotation using a protein-coding gene location file in build 38 obtained from the NCBI site, including transcription start site and end site (https://cncr.nl/research/magma/). We adopted an annotation window of 10 kb before the transcription start site and 10 kb after the end site to include SNPs around the gene. Then, genes were prioritized using the gene annotation file and the meta-analysis summary statistic, where genotype data from the European population in the phase 3 1000 Genomes project served as reference data, and a uniform sample size was specified.[35] We identified significant gene associations after Bonferroni correction using the number of annotated genes.

Human inner ear gene expression

Single-cell RNA-sequencing data of the human inner ear were obtained from the human inner ear atlas[47] and accessed through the gEAR Dashboard (https://umgear.org/). Processed gene expression matrices and corresponding cell type annotations provided by the atlas were downloaded and imported into R (version 4.4.2) for downstream visualization. Dot plots were generated using the DotPlot function in Seurat (version 5.3.1) to visualize the proportion of cells expressing selected genes and the average scaled expression level across annotated human inner ear cell types.

Genetic correlation

We estimated genetic correlations using LDSC.[39] Data processing and LD score calculation were discussed under heritability estimate. To assess shared genetic architecture between MD and other traits, we performed pairwise genetic correlation analysis using the ldsc.py –rg command. We processed summary statistics for 18 external GWAS summary statistics using the same pipeline and estimated genetic correlations using the same LD reference panel. For the list of external GWAS, please see Table S21.

Results

Meta-analysis identifies five independent genome-wide significant variants

We analyzed data across five biobanks: AoU[24,48] (n = 383,735, 841 MD case subjects), MVP[27] (n = 510,058, 2,306 case subjects), UKB[25] (n = 420,473, 1,220 case subjects), FinnGen[26] (n = 478,519, 3,680 case subjects), and BBJ[34] (n = 178,726, 1,290 case subjects) (Table 1). In aggregate, these datasets include 8,969 MD case subjects and 1,962,542 control subjects, corresponding to an overall prevalence of 0.46% in our study population. MD status was defined using phecode 386.1 or an equivalent phenotype mapped from ICD codes (H810, 3860, and 38599), with additional quality control steps applied as described under subjects and methods. To assess the contribution of common variants to MD genetic architecture and disease risk, we restricted analyses to variants with MAF > 1%, yielding 13,423,260 variants. We estimated that we had 80% power to detect an odds ratio (OR) of 1.9 for a variant at 1% frequency or an OR of 1.2 at 10% frequency (Figure S1). MD prevalence was higher in females (Figure 1A) and increased with age (Figures 1C and 1D),

Table 1. Summary of five contributing GWAS datasets

GWAS	Country; ancestry	Cases/control subjects	Case definition	Data type	Number of SNPs
All of Us	US; multi-ancestry	841/382,894	phecode 386.1	whole-genome sequenced	6,795,311
Million Veteran Program	US; European and Admixed American	2,306/507,752	phecode 386.1	genotyped and imputed	9,254,829
UK Biobank	UK; European	1,220/419,253	UKB phenocode 20002, coding 1421	genotyped and imputed	9,549,936
FinnGen	Finland; European	3,312/474,839	H81.0 (ICD-10); 3869 (ICD-9); 38599 (ICD-8)	genotyped and imputed	9,462,981
Biobank Japan	Japan; East Asian	1,290/177,436	phecode 386.1	genotyped and imputed	7,440,824

Each study is listed with its country of collection and ancestry filtering of case and control subjects, number of case and control subjects in each study, and number of SNPs after filtering for minor-allele frequency greater than 1%.

consistent with a prior report.[5] Across biobanks, 81.4% of MD case subjects were of European ancestry (Figure 1B), and case subjects were enriched for European ancestry within AoU (Figure S2).

We conducted a GWAS in AoU using Regenie[48] and then performed a fixed-effects meta-analysis with summary statistics from the four other studies[24–27,34] using the inverse-variance weighted scheme in METAL.[37] For each study, we filtered out variants with MAF <0.01 or minor-allele count <10. We estimated SNP-based heritability to be 7% (SE = 0.8%) on the observed scale using LDSC[39] applied to the meta-analyzed summary statistics. Minimal genomic inflation ($\lambda_{gc} = 1.01$) in the meta-analyzed result indicated negligible confounding before correction. We identified five independent genome-wide significant index SNPs ($p < 5 \times 10^{-8}$; Figure 2; Table 2), defined as the most significant variant within a 1 Mb window, clumped at $r^2 > 0.05$, and supported by at least one additional genome-wide significant SNP (Table S1). Three other loci were just below the significance threshold (subjects and methods; $5 \times 10^{-8} < p < 5 \times 10^{-7}$), and we highlight signals near *LMO4* and *ALDH1A2* due to their biological relevance (Tables S2 and S3).

Two independent signals at *EYA4* suggest a developmental contribution

We next examined the two independent association signals located 209 kb apart at *EYA4* (index SNPs rs11752136 and rs3777811, $r^2 = 0.003$; Table 2; Figure 3A). *EYA4* encodes a member of the evolutionarily conserved eyes absent (EYA)

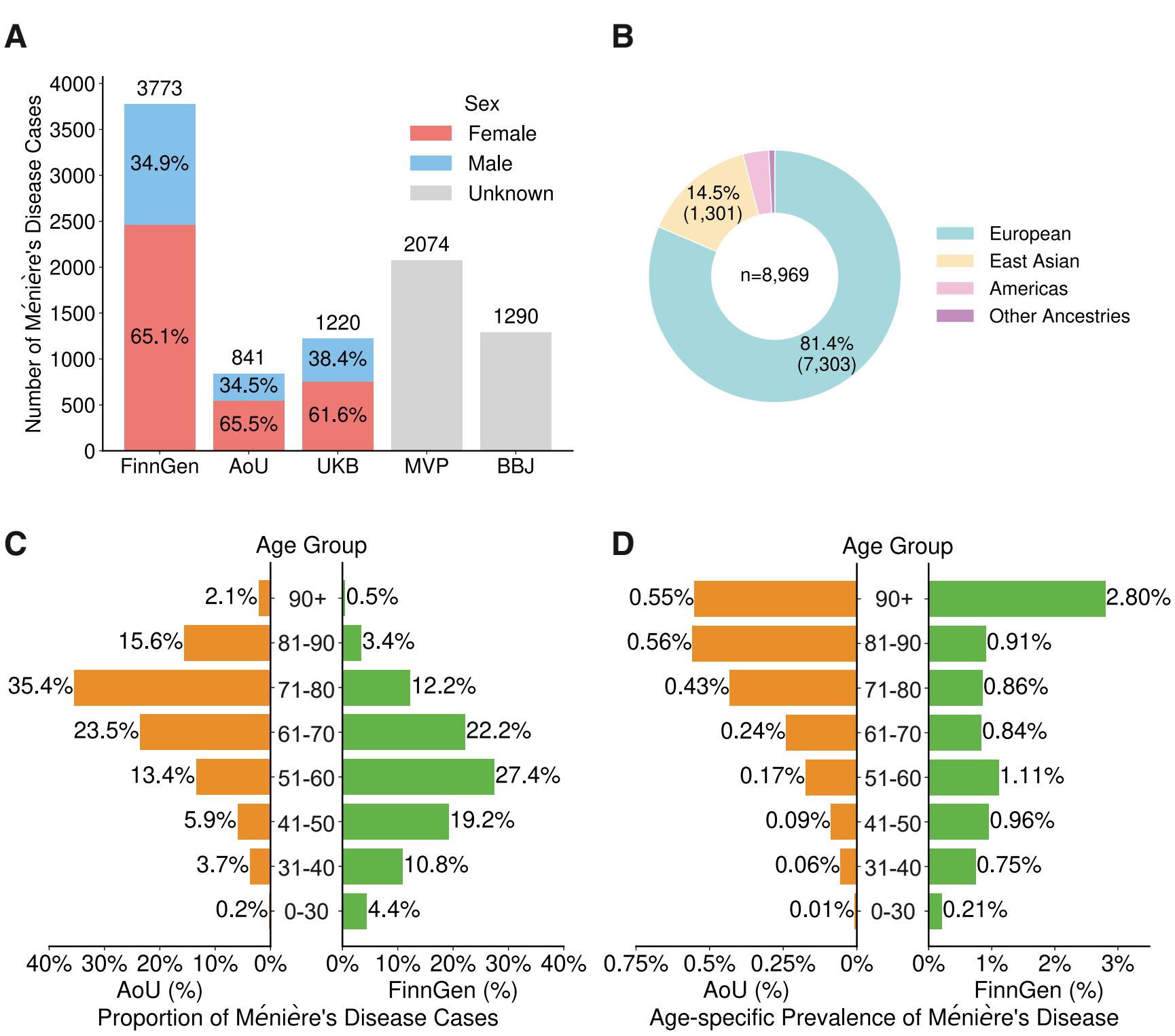

Figure 1. Demographics of MD case subjects
(A) Sex distribution of MD case subjects across FinnGen, AoU, and UKB. Information on the MVP and BBJ was not available.
(B) Genetically inferred ancestry distribution of MD case subjects across five contributing studies (subjects and methods). Other genetically inferred ancestries include African, South Asian, and Middle Eastern.
(C) Age distribution of MD case subjects in AoU and FinnGen, categorized by age bins, in proportion. Information on the MVP, the UKB, and BBJ was unavailable.
(D) Age-specific prevalence of MD case subjects in AoU and FinnGen.

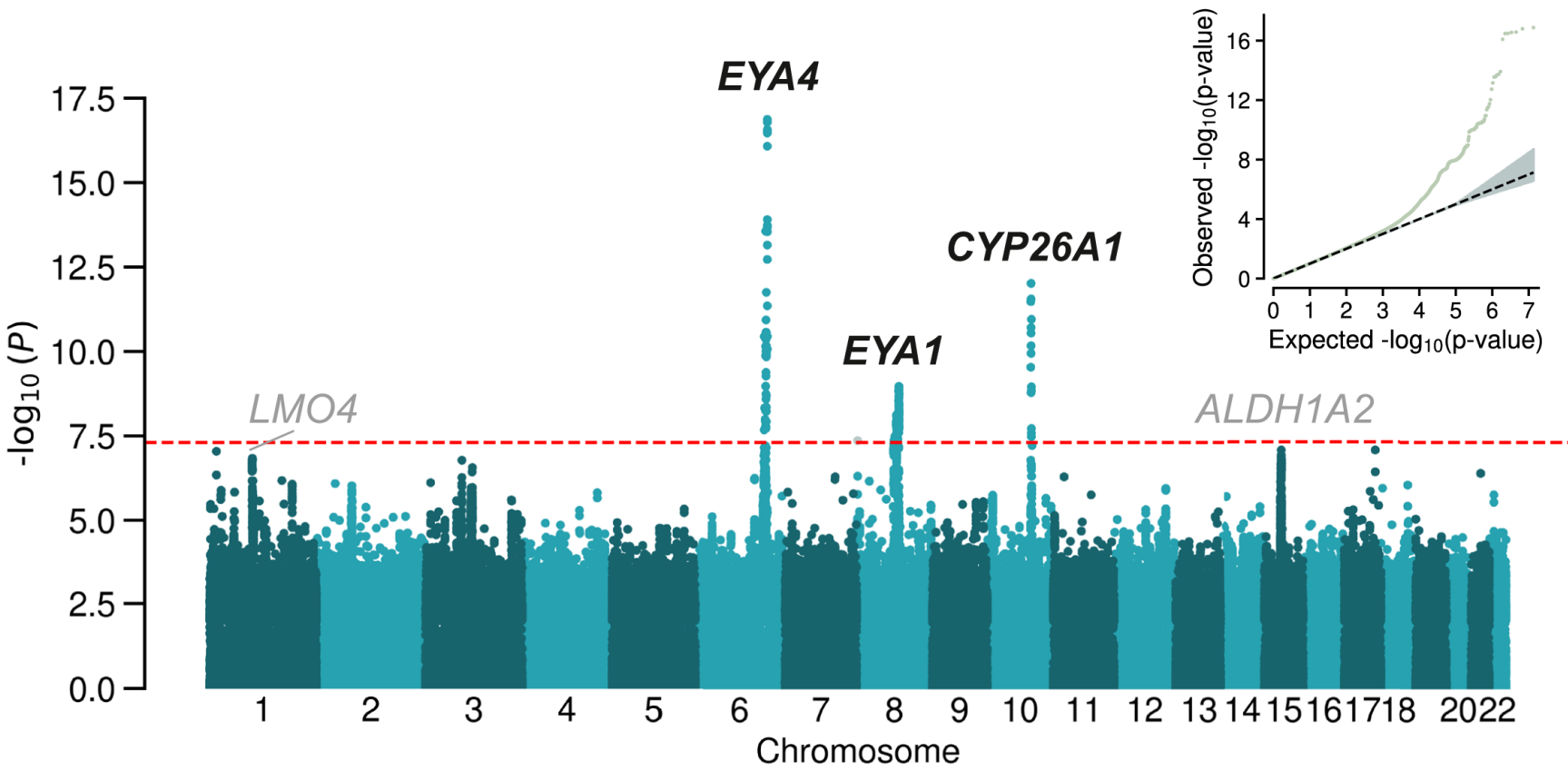

Figure 2. Manhattan plot of the meta-analysis
GC-corrected $-\log_{10}$ of p values from the meta-analysis of multi-ancestry MD GWASs. The red dashed line indicates the genome-wide significance threshold of 5×10^{-8}. Genome-wide significant variants, where no other significant variant is within 1 Mb, are plotted in gray. The nearest genes to the index SNPs are annotated in black. Additional loci discussed in the text are annotated in gray. Inset: quantile-quantile plot of the meta-analysis with genomic control applied.

protein family and functions as both a transcriptional coactivator and a phosphatase. *EYA4* has essential roles in auditory development and function, and loss-of-function mutations lead to autosomal-dominant SNHL (DFNA10).[49–53] Significant SNPs clumped with rs11752136 overlap candidate *cis*-regulatory elements located ~60 kb upstream of *EYA4* (Encode track, UCSC browser[54]), the *EYA4* transcription start site (TSS), the first intron, and an enhancer in intron 1. rs3777811 is located 155 kb downstream of the *EYA4* TSS in intron 3 (Figure 3A). Fine-mapping with SuSiE[44] resolved the region into two CSs corresponding to the two index SNPs; however, high LD precluded confident identification of a single causal variant in either region (Figure 3B; Tables S4–S6). *EYA4* shows a strong gene-level association with MD using MAGMA[46] ($p = 3.3 \times 10^{-10}$; Table S10).

EYA4 is expressed in sensory and non-sensory cell types of the cochlea and vestibular system at fetal and adult stages, according to single-cell RNA sequencing (RNA-seq) data from the human inner ear (Figure 4). Although eQTL data are not currently available for the inner ear,[45] rs3777811 is an eQTL for *EYA4* in several brain tissues, with the MD risk allele associated with increased *EYA4* expression (Table S11), whereas weak LD proxies of rs11752136 (rs971589, $r^2 = 0.48$) are eQTLs for *EYA4* across brain, heart, and tibial nerve tissues (Table S11). Phenome-wide analysis showed that rs3777811 (G allele) is associated with increased risk of SNHL, tinnitus, and general ear disorders, core symptoms that are genetically correlated with MD (Figures 5A and 5B), as well as decreased risk of sleep apnea (Table S16). Notably, rs3777811 is distinct from, and not in LD with, the previously identified coding variant rs9493627, which is associated with SNHL.[55] In contrast, rs11752136 is an MD-specific signal and shows no association with related phenotypes (Table S15). Together, these complementary signals suggest distinct regulatory mechanisms, one shared with SNHL and one specific to MD. Functional characterization of these two variants may help identify molecular mechanisms that are both specific to MD and shared with related disorders.

Two independent signals at *EYA1* further emphasize the role of development

At *EYA1*, we found another two independent index SNPs, rs2639899 and rs145240113 ($r^2 = 1 \times 10^{-4}$; Table 2; Figure 3A). rs2639899 lies 192 kb downstream of *EYA1* in a candidate *cis*-regulatory element within a genomic region on chromosome 8 that also features a cluster of five ultraconserved non-coding elements (UCNEBase track, UCSC Genome Browser[54]). rs145240113 is located 35 kb upstream of the *EYA1* TSS. Fine-mapping delineated two CSs corresponding to these index SNPs, though again, extensive local LD precluded identification of causal variants (Figures 3A and 3B; Tables S4, S7, and S8).

EYA1's association with MD reached gene-level significance (MAGMA $p = 1.3 \times 10^{-5}$; Table S10), and similar to *EYA4*, is expressed in the human inner ear at fetal and

Table 2. Five genome-wide significant index SNP associations

RsID	Location (chr:position)	Effect allele (EA)	Frequency of EA in 1000 Genomes EUR	Odds ratio	*p* Value	Closest gene
rs11752136	6:133184618	A	0.23	0.852	2.71×10^{-14}	*EYA4*
rs3777811	6:133394072	G	0.09	1.246	1.34×10^{-17}	*EYA4*
rs2639899	8:71007227	C	0.47	0.905	7.25×10^{-9}	*EYA1*
rs145240113	8:71583477	G	0.10	0.852	1.08×10^{-9}	*EYA1*
rs61861121	10:93191324	A	0.09	1.197	9.51×10^{-13}	*CYP26A1*

Listed are rsID, location in hg38, effect allele, frequency of EA in 1000 Genomes EUR population, odds ratio, *p* value, and closest gene by distance.

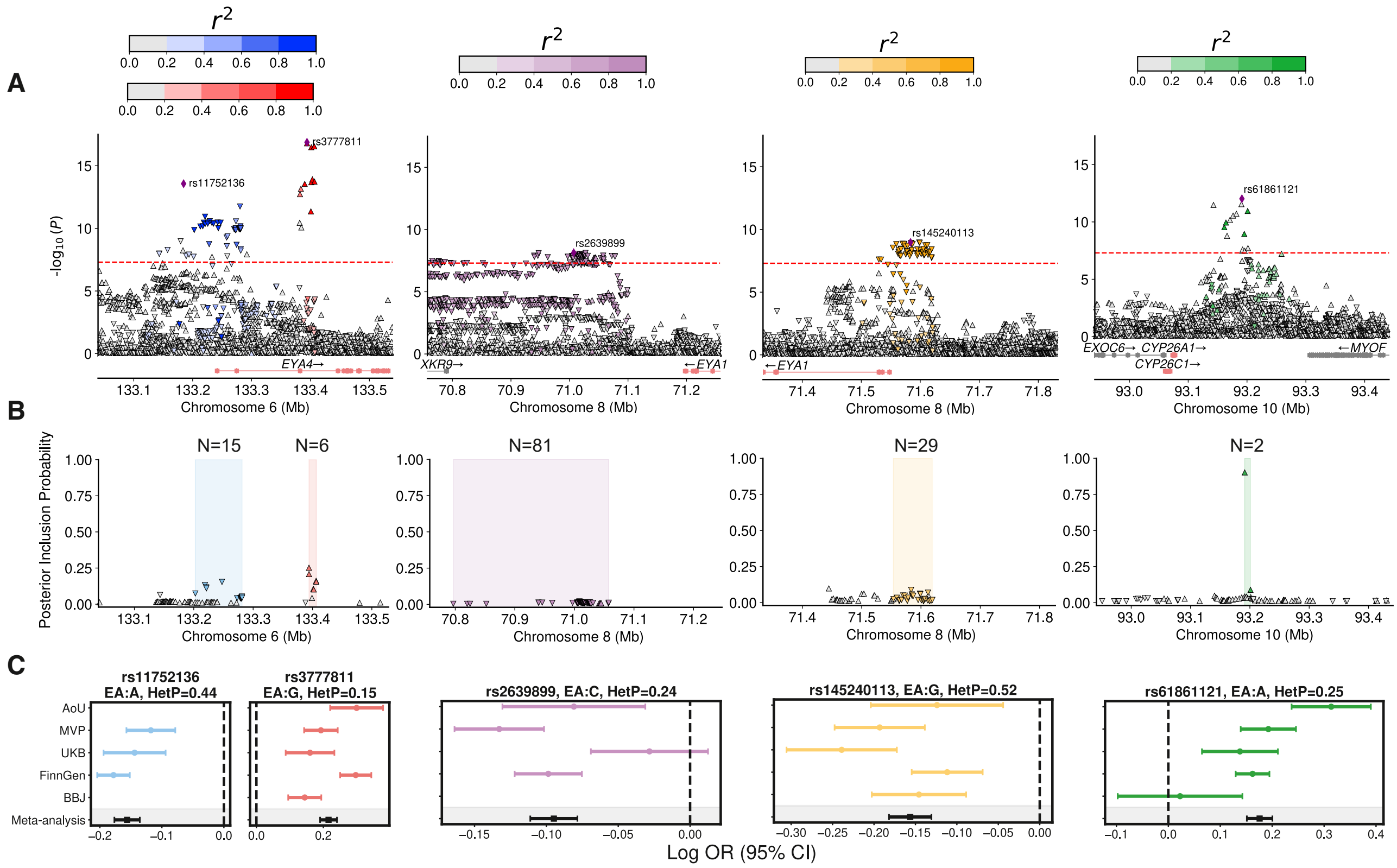

Figure 3. Characterization of genome-wide significant loci for MD

(A) $-\log_{10} p$ values from the meta-analysis plotted against genomic positions for each index SNP region. The window size of the plots is 500 kb. Genes of interest are colored in red along the x axis. A red dotted line denotes genome-wide significance ($p < 5 \times 10^{-8}$).

(B) Posterior inclusion probabilities (PIPs) from SuSiE fine-mapping plotted against genomic position for each index SNP region. Shading denotes the start and end of CSs, and the number of SNPs in the CS is annotated. SNPs with PIP <1% not shown.

(C) Forest plots of ORs as OR for each GWAS study and meta-analysis with 95% confidence intervals on the x axis for each index SNP. For each index SNP, effective allele and heterogeneity p values are included in the title.

adult stages (Figure 4). *EYA1* encodes a homologous dual-functioning transcriptional regulator and phosphatase, and its haploinsufficiency causes branchio-oto-renal (BOR) syndrome, a congenital disorder characterized by malformations of the outer, middle, and inner ear; hearing loss; and renal anomalies.[57–59] Although *EYA1* and *EYA4* share similar phosphatase substrates,[36] *EYA1* functions earlier in otic development than *EYA4*, with *EYA1*-deficient ears arresting at the otic vesicle stage.[60]

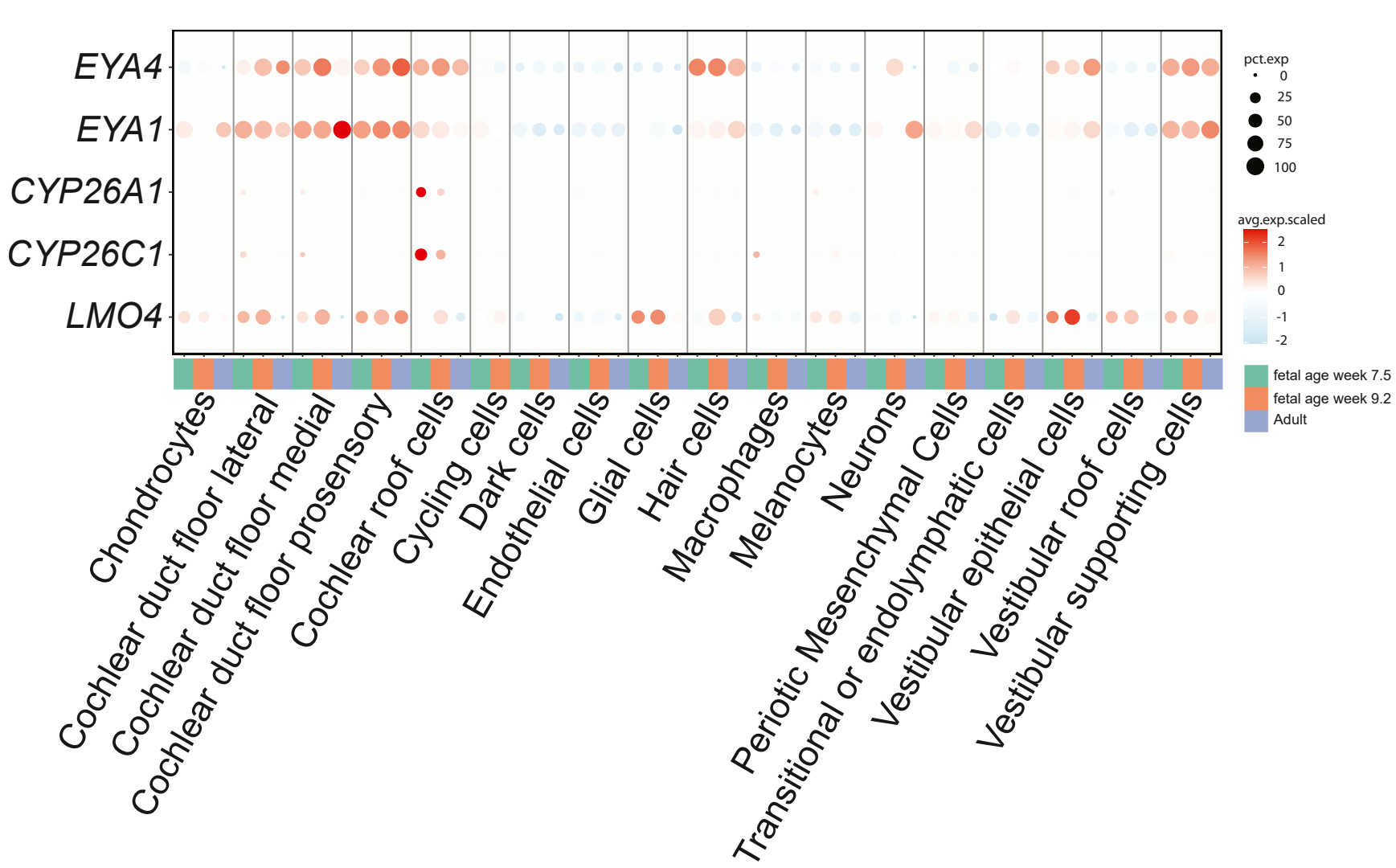

Figure 4. Dotplot of single-cell expression from human inner ear single-cell RNA-seq across developmental stages

The dot plot shows the expression of *EYA4*, *EYA1*, *CYP26A1*, *CYP26C1*, and *LMO4* across diverse human inner ear cell types at three developmental stages (fetal week 7.5, fetal week 9.2, and adult). Dot size represents the percentage of expressing cells, and color indicates scaled average expression. Data were obtained from the human inner ear atlas[47] via the gEAR dashboard.[56]

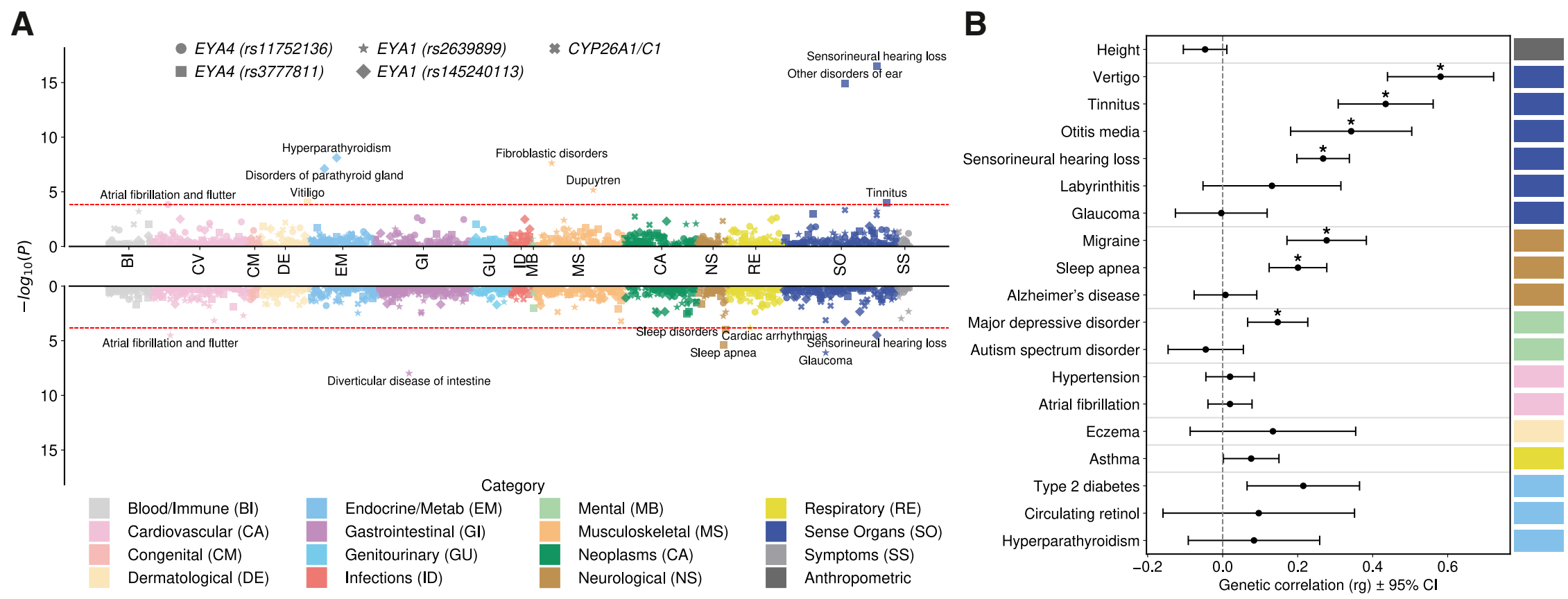

Figure 5. Phenotypic characterization
(A) Phenome-wide associations from harmonized FinnGen, MVP, and UKB GWAS results of the five index SNPs across disease categories are plotted. The red dotted line denotes the Bonferroni-corrected p value threshold. The top plot indicates the same direction of effect as MD, while the opposite direction is indicated in the bottom plot. Phenotypes with significant association are denoted.
(B) Forest plot of genetic correlation estimates of MD between phenotypes across categories. An asterisk denotes Bonferroni-corrected significance.

rs2639899 is an eQTL for *EYA1* in esophageal mucosa. Consistent with the effect of rs3777811 on *EYA4*, the MD risk allele of rs2639899 is associated with increased *EYA1* expression (Table S12). Besides MD, the risk-increasing allele of rs2639899 (T allele) is associated with several phenotypes, including reduced risk of glaucoma, while the risk-increasing allele of rs145240113 (A allele) is associated with increased risk of hyperparathyroidism and decreased risk of SNHL (Figure 5A; Tables S17 and S18).

An association at *LMO4* is consistent with its developmental role in mice

We observed a suggestive association at rs1199565, located 101 kb downstream of *LMO4* ($p = 1.46 \times 10^{-7}$; Table S2; Figure S3) and annotated as an eQTL in subcutaneous adipose tissue (Table S13). Although below genome-wide significance, this signal is noteworthy given the established role of *LMO4* in vestibular and cochlear morphogenesis; loss of *Lmo4* expression in mice disrupts inner ear development and impairs balance.[61,62] In the human fetal inner ear, single-cell RNA-seq data show strong *LMO4* expression in vestibular epithelial and cochlear duct floor cells (Figure 4), suggesting a conserved developmental function. Together with the *EYA4* and *EYA1* loci, this locus reinforces a broader theme in which dysregulation of genes guiding inner ear formation contributes to MD susceptibility.

The *CYP26A1/C1* locus implicates retinoic acid signaling

On chromosome 10, the index SNP rs61861121 lies downstream of *CYP26A1*, *CYP26C1*, *EXOC6*, and *MYOF* (Table 2; Figures 3A and 3B). Fine-mapping identified rs61861121 as a causal SNP within a two-variant 95% CS, with a PIP of 0.90 (Figure 3B; Tables S4 and S9). rs61861121 is not an eQTL for any nearby genes, and none reached gene-level significance in MAGMA[46] (*CYP26A1* $p = 0.03$; *CYP26C1* $p = 0.05$, *EXOC6* $p = 0.12$, *MYOF* $p = 0.05$; Table S10), leaving the causal gene unresolved. Nonetheless, functional considerations suggest that *CYP26A1/C1* are plausible candidates. Both genes encode enzymes that degrade retinoic acid to maintain spatial gradients during embryogenesis, a process essential for patterning of the retina, hindbrain, and otic placode.[63–66] The functional paralog of *CYP26A1/C1* in mice, *Cyp26b1*, has a critical role in vestibular sensory epithelium formation by shaping retinoic acid availability in the embryonic inner ear.[67] The vestibular structures affected are responsible for detecting head motion, directly relevant to MD. Although rs61861121 shows no association with other phenotypes after Bonferroni correction (Figure 5A), a more lenient threshold (subjects and methods) suggested associations with multiple eye and ear phenotypes, including glaucoma, peripheral retinal degeneration, and SNHL (Table S19). This convergence suggests a role for retinoic acid signaling in a broader category of fluid tension disorders spanning glaucoma, intracranial hypertension, and MD.[68] The potential role for retinoic acid signaling is further supported by a suggestive association at *ALDH1A2* (rs6493979; $p = 8.6 \times 10^{-8}$; Table S2; Figure S3), which is MAGMA significant ($p = 3.07 \times 10^{-7}$; Table S10) and has moderate expression in vestibular epithelial cells (Figure S4). *ALDH1A2 is* directly involved in retinoic acid synthesis[69–72] and associated with cerebral ventricle volume,[73] which, again, points to disruption of retinoic acid metabolism in MD etiology and a link with other fluid tension disorders.

Discussion

In this study, we report a large-scale GWAS of MD, integrating data from nearly two million individuals across five major biobanks. An observed-scale SNP heritability

of 0.07 indicates a measurable but modest contribution of common variants to disease risk. Common variation in genes associated with familial MD does not appear to make a substantial contribution to sporadic cases. We identify five genome-wide significant loci with functional and phenotypic evidence implicating developmental regulation of the inner ear and retinoic acid-mediated signaling. These findings expand our understanding of the genetic basis of MD beyond familial rare variants and point to biological pathways important in disease.

The most robust associations map to *EYA4* and *EYA1*, transcriptional regulators essential for otic placode and inner ear development.[51–53,58–60] Both loci harbor two independent non-coding signals. Two of the four index SNPs, rs11752136 at *EYA4* and rs145240113 at *EYA1*, act as eQTLs in various tissues, and MD risk alleles of both are associated with increased expression of *EYA4* and *EYA1* (Tables S11 and S12). Meanwhile, a significant association, rs3836957, located at *EYA4* and clumped with rs11752136, was identified as an enhancer (Table S20). These patterns suggest that MD-associated alleles modulate gene expression rather than causing loss of function. Expression analysis of human inner ear single-cell RNA-seq data further supports the relevance of these genes; both *EYA4* and *EYA1* are expressed in vestibular and cochlear epithelial cell types during fetal and adult stages, while *LMO4* is expressed in vestibular and cochlear duct floor cells during fetal stages (Figure 4). These findings converge on developmental dysregulation of sensory epithelia as a shared mechanism linking MD to congenital and acquired hearing disorders. Consistent with this model, *EYA4* variants have been implicated in autosomal-dominant SNHL,[49,50,53] pathogenic *EYA1* variants are causal in BOR syndrome,[59] and loss of *LMO4* expression leads to maldevelopment of the mouse inner ear.[57,58,61,62] The extension of these developmental pathways to MD, typically a late-onset, sporadic condition,[2,5] suggests that subtle regulatory perturbations in the expression of developmental genes may predispose to adult vestibular and auditory dysfunction, in agreement with a recently proposed model of MD pathophysiology.[74] Alternatively, as at least some MD risk genes (*EYA4* and *EYA1*) are also expressed in the adult inner ear, they may also serve homeostatic roles in maintaining inner ear function.

A complementary biological theme arises from the association near *CYP26A1/C1* and the suggestive signal at *ALDH1A2*. These genes encode enzymes that, respectively, degrade and synthesize retinoic acid (RA), maintaining local RA gradients essential for hindbrain and otic patterning.[64–66,75–78] Fine-mapping highlights rs61861121 as a likely causal variant with high posterior probability, pointing to regulatory perturbation of *CYP26A1/C1* rather than protein-altering effects. In mice, the paralog *Cyp26b1* controls vestibular sensory epithelium formation through spatial RA regulation, directly linking this pathway to balance.[67] These associations align with prior hypotheses connecting RA metabolism to endolymphatic homeostasis and provide a developmental-to-physiological bridge for MD pathogenesis.[74,75]

Phenome-wide association analyses and genome-wide genetic correlations highlight the intersection between MD and related sensory or neurological traits. The strongest polygenic overlaps are observed with vertigo, tinnitus, and SNHL, which are core symptoms of MD. We also observe a positive genetic correlation with migraine, a comorbidity of MD that may share vascular or neurogenic mechanisms.[79] However, despite a positive genetic correlation, GWAS signals for vertigo and migraine do not overlap with MD,[80,81] indicating that the largest effects are not shared. We also find no evidence that common variants at genes associated with familial MD contribute to risk of sporadic MD. On the other hand, we find overlap in GWAS signals but low genetic correlations for fluid-tension-related traits, including glaucoma, suggesting a shared biological pathway, albeit one that only explains a small proportion of risk.

To contextualize our common-variant findings relative to prior rare-variant and familial studies, common variants at previously reported genes associated with familial MD,[12–16,21] including *OTOG*, *FAM136A*, and *DTNA*, do not contribute substantially to sporadic MD risk at genome-wide significance (Figure S5; Table S23). Additionally, no gene reached exome-wide significance under rare-variant burden testing in UKB exome data,[82] although *OTOG* showed a nominal association (burden $p = 3.7 \times 10^{-3}$ for protein-truncating variants). However, we do not interpret these as refuting prior findings given the distinct study designs; population-level GWAS or burden tests have limited power to detect rare variants identified in familial studies. Instead, these results suggest that common regulatory variation and rare coding variants represent complementary layers of genetic risk for MD.

This study has several limitations. First, MD diagnosis across contributing biobanks was based on ICD codes or equivalent phecodes. MD is a clinically complex disorder with overlapping symptoms, including vertigo, fluctuating SNHL, tinnitus, and aural fullness,[1] and, diagnostic criteria may vary across healthcare systems. This limitation is inherent to large-scale biobank studies that rely on EHRs, and case/control misclassification is expected to bias effect size estimates toward zero and reduce statistical power. Second, for four of the five contributing biobanks, we relied on previously published summary statistics rather than individual-level data, limiting our ability to apply uniform quality control. Nonetheless, lack of effect heterogeneity across cohorts at index SNPs (Figure 3C) provides reassurance that the identified associations are robust to study-specific biases, while the lack of overlap with the GWAS of vertigo suggests that our signals are specific to MD.

Together, our results establish MD as a polygenic disorder influenced by regulatory variation in genes governing

inner ear development and RA signaling. These factors only explain a small fraction of risk, and it remains to be seen how they interact with other genetic factors or environmental triggers. These findings lay the groundwork for future functional studies using human inner ear organoids and *in vivo* models to test how subtle dysregulation of pathways involving *EYA1/4* and *CYP26A1/C1* pathways affects sensory epithelium formation and fluid balance. Beyond mechanistic insight, this work provides a foundation for constructing polygenic risk models and exploring comorbidity across sensory and neurological disorders. Expanding these analyses to larger and more ancestrally diverse cohorts and integrating inner ear-specific regulatory eQTL data will be the next steps toward a comprehensive understanding of MD genetics and pathology and its place within the broader landscape of auditory and vestibular disease.

Data and code availability

Summary statistics for the meta-analysis have been deposited in the GWAS catalog under accession number GCST90809428.

Acknowledgments

We gratefully acknowledge All of Us participants for their contributions, without whom this research would not have been possible. This research was carried out in accordance with the guidelines of the All of Us Research program. We also thank the National Institutes of Health's All of Us Research Program for making available the participant data examined in this study. This research is based on data from the Million Veteran Program, Office of Research and Development, Veterans Health Administration. We thank the Veterans who generously participated in the Million Veteran Program and made this research possible. We acknowledge the Pan-UK Biobank team and the UK Biobank participants and staff for making this resource publicly available. We also want to acknowledge the participants and investigators of the FinnGen study. We express our gratitude to all participants of the BioBank Japan Project and the medical coordinators of the cooperating hospitals for collecting samples and clinical information. The use of biobanks is in accordance with the ethical standards of the responsible committee, and proper informed consent was obtained. This work was supported by National Institute on Aging R01AG085518 (to B.P.), National Institute of Mental Health R01MH115676 (to B.P.), National Institute of General Medical Sciences R35GM133708 (to I.M.), and the National Institute on Deafness and Other Communication Disorders R01DC021475 (to D.J.E.). The content is solely the responsibility of the authors and does not necessarily represent the official views of the National Institutes of Health.

Author contributions

I.M. and D.J.E. conceived the project. I.M., B.P., D.J.E., and Z.S. designed experiments. Z.S. performed experiments and statistical analyses with assistance from R.M., X.L., Z.(E.)Z., S.L., and A.O.F.-C. J.L. QCed the genomic data in the All of Us Research Program. S.C. generated Figure 4. I.M., D.J.E., and B.P. supervised the project. Z.S., I.M., D.J.E., and B.P. wrote the paper with feedback from all authors.

Declaration of interests

The authors declare no competing interests.

Declaration of generative AI and AI-assisted technologies in the writing process

During the preparation of this work, ChatGPT was used to improve grammar and readability in the method section. After using this tool/service, the authors reviewed and edited the content as needed and take full responsibility for the content of the publication.

Supplemental information

Supplemental information can be found online at https://doi.org/10.1016/j.ajhg.2026.05.011.

Received: February 9, 2026
Accepted: May 22, 2026
Published: June 16, 2026

References

1. Minor, L.B., Schessel, D.A., and Carey, J.P. (2004). Ménière's disease. Curr. Opin. Neurol. *17*, 9–16.
2. Sajjadi, H., and Paparella, M.M. (2008). Meniere's disease. Lancet *372*, 406–414. https://doi.org/10.1016/S0140-6736(08)61161-7.
3. Pyykkö, I., Nakashima, T., Yoshida, T., Zou, J., and Naganawa, S. (2013). Ménière's disease: a reappraisal supported by a variable latency of symptoms and the MRI visualisation of endolymphatic hydrops. BMJ Open *3*, e001555. https://doi.org/10.1136/bmjopen-2012-001555.
4. Merchant, S.N., Adams, J.C., and Nadol, J.B., Jr. (2005). Pathophysiology of Ménière's Syndrome: Are Symptoms Caused by Endolymphatic Hydrops? Otol. Neurotol. *26*, 74–81.
5. Nakashima, T., Pyykkö, I., Arroll, M.A., Casselbrant, M.L., Foster, C.A., Manzoor, N.F., Megerian, C.A., Naganawa, S., and Young, Y.-H. (2016). Meniere's disease. Nat. Rev. Dis. Primer *2*, 1–18. https://doi.org/10.1038/nrdp.2016.28.
6. Tyrrell, J.S., Whinney, D.J.D., Ukoumunne, O.C., Fleming, L.E., and Osborne, N.J. (2014). Prevalence, Associated Factors, and Comorbid Conditions for Ménière's Disease. Ear Hear. *35*, e162–e169. https://doi.org/10.1097/AUD.0000000000000041.
7. Harris, J.P., and Alexander, T.H. (2010). Current-Day Prevalence of Ménière's Syndrome. Audiol. Neurootol. *15*, 318–322. https://doi.org/10.1159/000286213.
8. Havia, M., Kentala, E., and PyykkÖ, I. (2005). Prevalence of Menière's Disease in General Population of Southern Finland. Otolaryngol. Neck Surg. *133*, 762–768. https://doi.org/10.1016/j.otohns.2005.06.015.
9. Ohmen, J.D., White, C.H., Li, X., Wang, J., Fisher, L.M., Zhang, H., Derebery, M.J., and Friedman, R.A. (2013). Genetic Evidence for an Ethnic Diversity in the Susceptibility to Ménière's Disease. Otol. Neurotol. *34*, 1336–1341. https://doi.org/10.1097/MAO.0b013e3182868818.

10. Yang, T.-H., Xirasagar, S., Cheng, Y.-F., Wu, C.-S., Kuo, N.-W., and Lin, H.-C. (2021). Peripheral Vestibular Disorders: Nationwide Evidence From Taiwan. Laryngoscope *131*, 639–643. https://doi.org/10.1002/lary.28877.
11. Parra-Perez, A.M., and Lopez-Escamez, J.A. (2023). Types of Inheritance and Genes Associated with Familial Meniere Disease. J. Assoc. Res. Otolaryngol. *24*, 269–279. https://doi.org/10.1007/s10162-023-00896-0.
12. Gallego-Martinez, A., Requena, T., Roman-Naranjo, P., and Lopez-Escamez, J.A. (2019). Excess of Rare Missense Variants in Hearing Loss Genes in Sporadic Meniere Disease. Front. Genet. *10*, 76. https://doi.org/10.3389/fgene.2019.00076.
13. Gallego-Martinez, A., Requena, T., Roman-Naranjo, P., May, P., and Lopez-Escamez, J.A. (2020). Enrichment of damaging missense variants in genes related with axonal guidance signalling in sporadic Meniere's disease. J. Med. Genet. *57*, 82–88. https://doi.org/10.1136/jmedgenet-2019-106159.
14. Fisch, K.M., Rosenthal, S.B., Mark, A., Sasik, R., Nasamran, C.A., Clifford, R., Derebery, M.J., Boussaty, E., Jepsen, K., Harris, J., and Friedman, R.A. (2024). The genomic landscape of Ménière's disease: a path to endolymphatic hydrops. BMC Genom. *25*, 646. https://doi.org/10.1186/s12864-024-10552-3.
15. Roman-Naranjo, P., Moleon, M.D.C., Aran, I., Escalera-Balsera, A., Soto-Varela, A., Bächinger, D., Gomez-Fiñana, M., Eckhard, A.H., and Lopez-Escamez, J.A. (2021). Rare coding variants involving MYO7A and other genes encoding stereocilia link proteins in familial meniere disease. Hear. Res. *409*, 108329. https://doi.org/10.1016/j.heares.2021.108329.
16. Requena, T., Cabrera, S., Martín-Sierra, C., Price, S.D., Lysakowski, A., and Lopez-Escamez, J.A. (2015). Identification of two novel mutations in FAM136A and DTNA genes in autosomal-dominant familial Meniere's disease. Hum. Mol. Genet. *24*, 1119–1126. https://doi.org/10.1093/hmg/ddu524.
17. Roman-Naranjo, P., Gallego-Martinez, A., Soto-Varela, A., Aran, I., Moleon, M.d.C., Espinosa-Sanchez, J.M., Amor-Dorado, J.C., Batuecas-Caletrio, A., Perez-Vazquez, P., and Lopez-Escamez, J.A. (2020). Burden of Rare Variants in the OTOG Gene in Familial Meniere's Disease. Ear Hear. *41*, 1598–1605. https://doi.org/10.1097/AUD.0000000000000878.
18. Klockars, T., and Kentala, E. (2007). Inheritance of Ménière's Disease in the Finnish Population. Arch. Otolaryngol. Neck Surg. *133*, 73–77. https://doi.org/10.1001/archotol.133.1.73.
19. Morrison, A.W., and Johnson, K.J. (2002). Genetics (molecular biology) and Meniere's disease. Otolaryngol. Clin. North Am. *35*, 497–516. https://doi.org/10.1016/S0030-6665(02)00018-X.
20. Morrison, A.W., Bailey, M.E.S., and Morrison, G.a.J. (2009). Familial Ménière's disease: clinical and genetic aspects. J. Laryngol. Otol. *123*, 29–37. https://doi.org/10.1017/S0022215108002788.
21. Escalera-Balsera, A., Robles-Bolivar, P., Parra-Perez, A.M., Murillo-Cuesta, S., Chua, H.C., Rodríguez-de la Rosa, L., Contreras, J., Domarecka, E., Amor-Dorado, J.C., Soto-Varela, A., et al. (2025). A rare haplotype of the GJD3 gene segregating in familial Meniere's disease interferes with connexin assembly. Genome Med. *17*, 4. https://doi.org/10.1186/s13073-024-01425-1.
22. Uffelmann, E., Huang, Q.Q., Munung, N.S., de Vries, J., Okada, Y., Martin, A.R., Martin, H.C., Lappalainen, T., and Posthuma, D. (2021). Genome-wide association studies. Nat. Rev. Methods Primers *1*, 59. https://doi.org/10.1038/s43586-021-00056-9.
23. Bycroft, C., Freeman, C., Petkova, D., Band, G., Elliott, L.T., Sharp, K., Motyer, A., Vukcevic, D., Delaneau, O., O'Connell, J., et al. (2018). The UK Biobank resource with deep phenotyping and genomic data. Nature *562*, 203–209. https://doi.org/10.1038/s41586-018-0579-z.
24. (2024). The All of Us Research Program Genomics Investigators. Genomic data in the All of Us Research Program. Nature *627*, 340–346. https://doi.org/10.1038/s41586-023-06957-x.
25. Karczewski, K.J., Gupta, R., Kanai, M., Lu, W., Tsuo, K., Wang, Y., Walters, R.K., Turley, P., Callier, S., Shah, N.N., et al. (2025). Pan-UK Biobank genome-wide association analyses enhance discovery and resolution of ancestry-enriched effects. Nat. Genet. *57*, 2408–2417. https://doi.org/10.1038/s41588-025-02335-7.
26. Kurki, M.I., Karjalainen, J., Palta, P., Sipilä, T.P., Kristiansson, K., Donner, K.M., Reeve, M.P., Laivuori, H., Aavikko, M., Kaunisto, M.A., et al. (2023). FinnGen provides genetic insights from a well-phenotyped isolated population. Nature *613*, 508–518. https://doi.org/10.1038/s41586-022-05473-8.
27. Verma, A., Huffman, J.E., Rodriguez, A., Conery, M., Liu, M., Ho, Y.-L., Kim, Y., Heise, D.A., Guare, L., Panickan, V.A., et al. (2024). Diversity and scale: Genetic architecture of 2068 traits in the VA Million Veteran Program. Science *385*, eadj1182. https://doi.org/10.1126/science.adj1182.
28. Nagai, A., Hirata, M., Kamatani, Y., Muto, K., Matsuda, K., Kiyohara, Y., Ninomiya, T., Tamakoshi, A., Yamagata, Z., Mushiroda, T., et al. (2017). Overview of the BioBank Japan Project: Study design and profile. J. Epidemiol. *27*, S2–S8. https://doi.org/10.1016/j.je.2016.12.005.
29. Zeggini, E., and Ioannidis, J.P. (2009). Meta-Analysis in Genome-Wide Association Studies. Pharmacogenomics *10*, 191–201. https://doi.org/10.2217/14622416.10.2.191.
30. Evangelou, E., and Ioannidis, J.P.A. (2013). Meta-analysis methods for genome-wide association studies and beyond. Nat. Rev. Genet. *14*, 379–389. https://doi.org/10.1038/nrg3472.
31. Li, X., Quick, C., Zhou, H., Gaynor, S.M., Liu, Y., Chen, H., Selvaraj, M.S., Sun, R., Dey, R., Arnett, D.K., et al. (2023). Powerful, scalable and resource-efficient meta-analysis of rare variant associations in large whole genome sequencing studies. Nat. Genet. *55*, 154–164. https://doi.org/10.1038/s41588-022-01225-6.
32. Gaziano, J.M., Concato, J., Brophy, M., Fiore, L., Pyarajan, S., Breeling, J., Whitbourne, S., Deen, J., Shannon, C., Humphries, D., et al. (2016). Million Veteran Program: A mega-biobank to study genetic influences on health and disease. J. Clin. Epidemiol. *70*, 214–223. https://doi.org/10.1016/j.jclinepi.2015.09.016.
33. Zhou, W., Bi, W., Zhao, Z., Dey, K.K., Jagadeesh, K.A., Karczewski, K.J., Daly, M.J., Neale, B.M., and Lee, S. (2022). SAIGE-GENE+ improves the efficiency and accuracy of set-based rare variant association tests. Nat. Genet. *54*, 1466–1469. https://doi.org/10.1038/s41588-022-01178-w.
34. Sakaue, S., Kanai, M., Tanigawa, Y., Karjalainen, J., Kurki, M., Koshiba, S., Narita, A., Konuma, T., Yamamoto, K., Akiyama, M., et al. (2021). A cross-population atlas of genetic associations for 220 human phenotypes. Nat. Genet. *53*, 1415–1424. https://doi.org/10.1038/s41588-021-00931-x.
35. Auton, A., Abecasis, G.R., Altshuler, D.M., Durbin, R.M., Abecasis, G.R., Bentley, D.R., Chakravarti, A., Clark, A.G., Donnelly, P., Eichler, E.E., et al. (2015). A global reference for human genetic variation. Nature *526*, 68–74. https://doi.org/10.1038/nature15393.

36. Bergström, A., McCarthy, S.A., Hui, R., Almarri, M.A., Ayub, Q., Danecek, P., Chen, Y., Felkel, S., Hallast, P., Kamm, J., et al. (2020). Insights into human genetic variation and population history from 929 diverse genomes. Science *367*, eaay5012. https://doi.org/10.1126/science.aay5012.
37. Willer, C.J., Li, Y., and Abecasis, G.R. (2010). METAL: fast and efficient meta-analysis of genomewide association scans. Bioinformatics *26*, 2190–2191. https://doi.org/10.1093/bioinformatics/btq340.
38. Perez, G., Barber, G.P., Benet-Pages, A., Casper, J., Clawson, H., Diekhans, M., Fischer, C., Gonzalez, J.N., Hinrichs, A.S., Lee, C.M., et al. (2025). The UCSC Genome Browser database: 2025 update. Nucleic Acids Res. *53*, D1243–D1249. https://doi.org/10.1093/nar/gkae974.
39. Bulik-Sullivan, B.K., Loh, P.-R., Finucane, H.K., Ripke, S., Yang, J., Patterson, N., Daly, M.J., Price, A.L., and Neale, B.M. (2015). LD Score regression distinguishes confounding from polygenicity in genome-wide association studies. Nat. Genet. *47*, 291–295. https://doi.org/10.1038/ng.3211.
40. Lee, S.H., Yang, J., Goddard, M.E., Visscher, P.M., and Wray, N.R. (2012). Estimation of pleiotropy between complex diseases using single-nucleotide polymorphism-derived genomic relationships and restricted maximum likelihood. Bioinformatics *28*, 2540–2542. https://doi.org/10.1093/bioinformatics/bts474.
41. Ojavee, S.E., Kutalik, Z., and Robinson, M.R. (2022). Liability-scale heritability estimation for biobank studies of low-prevalence disease. Am. J. Hum. Genet. *109*, 2009–2017. https://doi.org/10.1016/j.ajhg.2022.09.011.
42. Purcell, S., Neale, B., Todd-Brown, K., Thomas, L., Ferreira, M.A.R., Bender, D., Maller, J., Sklar, P., de Bakker, P.I.W., Daly, M.J., and Sham, P.C. (2007). PLINK: A Tool Set for Whole-Genome Association and Population-Based Linkage Analyses. Am. J. Hum. Genet. *81*, 559–575. https://doi.org/10.1086/519795.
43. McLaren, W., Gil, L., Hunt, S.E., Riat, H.S., Ritchie, G.R.S., Thormann, A., Flicek, P., and Cunningham, F. (2016). The Ensembl Variant Effect Predictor. Genome Biol. *17*, 122. https://doi.org/10.1186/s13059-016-0974-4.
44. Zou, Y., Carbonetto, P., Wang, G., and Stephens, M. (2022). Fine-mapping from summary data with the "Sum of Single Effects" model. PLoS Genet. *18*, e1010299. https://doi.org/10.1371/journal.pgen.1010299.
45. The Gtex Consortium (2020). The GTEx Consortium atlas of genetic regulatory effects across human tissues. Science *369*, 1318–1330. https://doi.org/10.1126/science.aaz1776.
46. de Leeuw, C.A., Mooij, J.M., Heskes, T., and Posthuma, D. (2015). MAGMA: Generalized Gene-Set Analysis of GWAS Data. PLoS Comput. Biol. *11*, e1004219. https://doi.org/10.1371/journal.pcbi.1004219.
47. Valk, W.H. van der, Beelen, E.S.A. van, Steinhart, M.R., Nist-Lund, C., Osorio, D., Groot, J.C.M.J. de, Sun, L., Benthem, P.P.G. van, Koehler, K.R., and Locher, H. (2023). A single-cell level comparison of human inner ear organoids with the human cochlea and vestibular organs. Cell Rep. *42*, 112623. https://doi.org/10.1016/j.celrep.2023.112623.
48. Mbatchou, J., Barnard, L., Backman, J., Marcketta, A., Kosmicki, J.A., Ziyatdinov, A., Benner, C., O'Dushlaine, C., Barber, M., Boutkov, B., et al. (2021). Computationally efficient whole-genome regression for quantitative and binary traits. Nat. Genet. *53*, 1097–1103. https://doi.org/10.1038/s41588-021-00870-7.
49. Abe, S., Takeda, H., Nishio, S.y., and Usami, S.i. (2018). Sensorineural hearing loss and mild cardiac phenotype caused by an EYA4 mutation. Hum. Genome Var. *5*, 23. https://doi.org/10.1038/s41439-018-0023-9.
50. Shinagawa, J., Moteki, H., Nishio, S.y., Ohyama, K., Otsuki, K., Iwasaki, S., Masuda, S., Oshikawa, C., Ohta, Y., Arai, Y., et al. (2020). Prevalence and clinical features of hearing loss caused by EYA4 variants. Sci. Rep. *10*, 3662. https://doi.org/10.1038/s41598-020-60259-0.
51. Depreux, F.F.S., Darrow, K., Conner, D.A., Eavey, R.D., Liberman, M.C., Seidman, C.E., and Seidman, J.G. (2008). *Eya4*-deficient mice are a model for heritable otitis media. J. Clin. Investig. *118*, 651–658. https://doi.org/10.1172/JCI32899.
52. Wang, L., Sewell, W.F., Kim, S.D., Shin, J.T., MacRae, C.A., Zon, L.I., Seidman, J.G., and Seidman, C.E. (2008). Eya4 regulation of Na+/K+-ATPase is required for sensory system development in zebrafish. Development *135*, 3425–3434. https://doi.org/10.1242/dev.012237.
53. Wayne, S., Robertson, N.G., DeClau, F., Chen, N., Verhoeven, K., Prasad, S., Tranebjärg, L., Morton, C.C., Ryan, A.F., Van Camp, G., et al. (2001). Mutations in the transcriptional activator EYA4 cause late-onset deafness at the DFNA10 locus. Hum. Mol. Genet. *10*, 195–200. https://doi.org/10.1093/hmg/10.3.195.
54. Casper, J., Speir, M.L., Raney, B.J., Perez, G., Nassar, L.R., Lee, C.M., Hinrichs, A.S., Gonzalez, J.N., Fischer, C., Diekhans, M., et al. (2026). The UCSC Genome Browser database: 2026 update. Nucleic Acids Res. *54*, D1331–D1335. https://doi.org/10.1093/nar/gkaf1250.
55. Wells, H.R.R., Freidin, M.B., Zainul Abidin, F.N., Payton, A., Dawes, P., Munro, K.J., Morton, C.C., Moore, D.R., Dawson, S.J., and Williams, F.M.K. (2019). GWAS Identifies 44 Independent Associated Genomic Loci for Self-Reported Adult Hearing Difficulty in UK Biobank. Am. J. Hum. Genet. *105*, 788–802. https://doi.org/10.1016/j.ajhg.2019.09.008.
56. Orvis, J., Gottfried, B., Kancherla, J., Adkins, R.S., Song, Y., Dror, A.A., Olley, D., Rose, K., Chrysostomou, E., Kelly, M.C., et al. (2021). gEAR: Gene Expression Analysis Resource portal for community-driven, multi-omic data exploration. Nat. Methods *18*, 843–844. https://doi.org/10.1038/s41592-021-01200-9.
57. Chang, E.H., Menezes, M., Meyer, N.C., Cucci, R.A., Vervoort, V.S., Schwartz, C.E., and Smith, R.J.H. (2004). Branchio-oto-renal syndrome: The mutation spectrum in EYA1 and its phenotypic consequences. Hum. Mutat. *23*, 582–589. https://doi.org/10.1002/humu.20048.
58. Xu, P.-X., Adams, J., Peters, H., Brown, M.C., Heaney, S., and Maas, R. (1999). Eya1-deficient mice lack ears and kidneys and show abnormal apoptosis of organ primordia. Nat. Genet. *23*, 113–117. https://doi.org/10.1038/12722.
59. Abdelhak, S., Kalatzis, V., Heilig, R., Compain, S., Samson, D., Vincent, C., Weil, D., Cruaud, C., Sahly, I., Leibovici, M., et al. (1997). A human homologue of the Drosophila eyes absent gene underlies Branchio-Oto-Renal (BOR) syndrome and identifies a novel gene family. Nat. Genet. *15*, 157–164. https://doi.org/10.1038/ng0297-157.
60. Zou, D., Erickson, C., Kim, E.-H., Jin, D., Fritzsch, B., and Xu, P.-X. (2008). Eya1 gene dosage critically affects the development of sensory epithelia in the mammalian inner ear. Hum. Mol. Genet. *17*, 3340–3356. https://doi.org/10.1093/hmg/ddn229.
61. Deng, M., Pan, L., Xie, X., and Gan, L. (2010). Requirement for *Lmo4* in the vestibular morphogenesis of mouse inner

ear. Dev. Biol. *338*, 38–49. https://doi.org/10.1016/j.ydbio.2009.11.003.

62. Deng, M., Luo, X.j., Pan, L., Yang, H., Xie, X., Liang, G., Huang, L., Hu, F., Kiernan, A.E., and Gan, L. (2014). LMO4 Functions As a Negative Regulator of Sensory Organ Formation in the Mammalian Cochlea. J. Neurosci. *34*, 10072–10077. https://doi.org/10.1523/JNEUROSCI.0352-14.2014.
63. Sakai, Y., Luo, T., McCaffery, P., Hamada, H., and Dräger, U.C. (2004). CYP26A1 and CYP26C1 cooperate in degrading retinoic acid within the equatorial retina during later eye development. Dev. Biol. *276*, 143–157. https://doi.org/10.1016/j.ydbio.2004.08.032.
64. White, R.J., and Schilling, T.F. (2008). How degrading: Cyp26s in hindbrain development. Dev. Dyn. *237*, 2775–2790. https://doi.org/10.1002/dvdy.21695.
65. Emoto, Y., Wada, H., Okamoto, H., Kudo, A., and Imai, Y. (2005). Retinoic acid-metabolizing enzyme Cyp26a1 is essential for determining territories of hindbrain and spinal cord in zebrafish. Dev. Biol. *278*, 415–427. https://doi.org/10.1016/j.ydbio.2004.11.023.
66. Abu-Abed, S., Dollé, P., Metzger, D., Beckett, B., Chambon, P., and Petkovich, M. (2001). The retinoic acid-metabolizing enzyme, CYP26A1, is essential for normal hindbrain patterning, vertebral identity, and development of posterior structures. Genes Dev. *15*, 226–240. https://doi.org/10.1101/gad.855001.
67. Ono, K., Keller, J., López Ramírez, O., González Garrido, A., Zobeiri, O.A., Chang, H.H.V., Vijayakumar, S., Ayiotis, A., Duester, G., Della Santina, C.C., et al. (2020). Retinoic acid degradation shapes zonal development of vestibular organs and sensitivity to transient linear accelerations. Nat. Commun. *11*, 63. https://doi.org/10.1038/s41467-019-13710-4.
68. Moodley, A., and Van Der Meyden, K. (2019). Could there be any merit in lumping primary open-angle glaucoma, idiopathic intracranial hypertension and Meniere's disease into a novel and discrete category of fluid tension disorders? Med. Hypotheses *132*, 109361. https://doi.org/10.1016/j.mehy.2019.109361.
69. Kim, H., Lapointe, J., Kaygusuz, G., Ong, D.E., Li, C., van de Rijn, M., Brooks, J.D., and Pollack, J.R. (2005). The Retinoic Acid Synthesis Gene ALDH1a2 Is a Candidate Tumor Suppressor in Prostate Cancer. Cancer Res. *65*, 8118–8124. https://doi.org/10.1158/0008-5472.CAN-04-4562.
70. Touma, S.E., Perner, S., Rubin, M.A., Nanus, D.M., and Gudas, L.J. (2009). Retinoid metabolism and ALDH1A2 (RALDH2) expression are altered in the transgenic adenocarcinoma mouse prostate model. Biochem. Pharmacol. *78*, 1127–1138. https://doi.org/10.1016/j.bcp.2009.06.022.
71. El Kares, R., Manolescu, D.C., Lakhal-Chaieb, L., Montpetit, A., Zhang, Z., Bhat, P.V., and Goodyer, P. (2010). A human *ALDH1A2* gene variant is associated with increased newborn kidney size and serum retinoic acid. Kidney Int. *78*, 96–102. https://doi.org/10.1038/ki.2010.101.
72. Beecroft, S.J., Ayala, M., McGillivray, G., Nanda, V., Agolini, E., Novelli, A., Digilio, M.C., Dotta, A., Carrozzo, R., Clayton, J., et al. (2021). Biallelic hypomorphic variants in ALDH1A2 cause a novel lethal human multiple congenital anomaly syndrome encompassing diaphragmatic, pulmonary, and cardiovascular defects. Hum. Mutat. *42*, 506–519. https://doi.org/10.1002/humu.24179.
73. Ge, Y.-J., Wu, B.-S., Zhang, Y., Chen, S.-D., Zhang, Y.-R., Kang, J.-J., Deng, Y.-T., Ou, Y.-N., He, X.-Y., Zhao, Y.-L., et al. (2023). Genetic architectures of cerebral ventricles and their overlap with neuropsychiatric traits. Nat. Hum. Behav. *8*, 164–180. https://doi.org/10.1038/s41562-023-01722-6.
74. Chari, D.A., Bose, A., Ramirez, K., Robles-Bolivar, P., Lin, K.-Y., Juliano, A.F., Rauch, S.D., and Eckhard, A.H. (2025). A modern conceptual framework for study and treatment of Meniere's disease. Front. Neurol. *16*, 1607435. https://doi.org/10.3389/fneur.2025.1607435.
75. Romand, R., Hashino, E., Dollé, P., Vonesch, J.-L., Chambon, P., and Ghyselinck, N.B. (2002). The retinoic acid receptors RARα and RARγ are required for inner ear development. Mech. Dev. *119*, 213–223. https://doi.org/10.1016/S0925-4773(02)00385-4.
76. Bok, J., Raft, S., Kong, K.-A., Koo, S.K., Dräger, U.C., and Wu, D.K. (2011). Transient retinoic acid signaling confers anterior-posterior polarity to the inner ear. Proc. Natl. Acad. Sci. *108*, 161–166. https://doi.org/10.1073/pnas.1010547108.
77. Frenz, D.A., Liu, W., Cvekl, A., Xie, Q., Wassef, L., Quadro, L., Niederreither, K., Maconochie, M., and Shanske, A. (2010). Retinoid signaling in inner ear development: A "Goldilocks" phenomenon. Am. J. Med. Genet. *152A*, 2947–2961. https://doi.org/10.1002/ajmg.a.33670.
78. Maier, E.C., and Whitfield, T.T. (2014). RA and FGF Signalling Are Required in the Zebrafish Otic Vesicle to Pattern and Maintain Ventral Otic Identities. PLoS Genet. *10*, e1004858. https://doi.org/10.1371/journal.pgen.1004858.
79. Kim, S.Y., Lee, C.H., Yoo, D.M., Kwon, M.J., Kim, J.H., Kim, J.-H., Park, B., Lee, H.-J., and Choi, H.G. (2022). Association Between Meniere Disease and Migraine. JAMA Otolaryngol. Neck Surg. *148*, 457–464. https://doi.org/10.1001/jamaoto.2022.0331.
80. Hautakangas, H., Winsvold, B.S., Ruotsalainen, S.E., Bjornsdottir, G., Harder, A.V.E., Kogelman, L.J.A., Thomas, L.F., Noordam, R., Benner, C., Gormley, P., et al. (2022). Genome-wide analysis of 102,084 migraine cases identifies 123 risk loci and subtype-specific risk alleles. Nat. Genet. *54*, 152–160. https://doi.org/10.1038/s41588-021-00990-0.
81. Skuladottir, A.T., Bjornsdottir, G., Nawaz, M.S., Petersen, H., Rognvaldsson, S., Moore, K.H.S., Olafsson, P.I., Magnusson, S.H., Bjornsdottir, A., Sveinsson, O.A., et al. (2021). A genome-wide meta-analysis uncovers six sequence variants conferring risk of vertigo. Commun. Biol. *4*, 1148. https://doi.org/10.1038/s42003-021-02673-2.
82. Wang, Q., Dhindsa, R.S., Carss, K., Harper, A.R., Nag, A., Tachmazidou, I., Vitsios, D., Deevi, S.V.V., Mackay, A., Muthas, D., et al. (2021). Rare variant contribution to human disease in 281,104 UK Biobank exomes. Nature *597*, 527–532. https://doi.org/10.1038/s41586-021-03855-y.

The American Journal of Human Genetics, Volume *113*

Supplemental information

Genome-wide analysis implicates

inner ear development in Ménière disease

Zhuozheng Shi, Ravi Mandla, Jingjing Li, Xinzhe Li, Zixuan (Eleanor) Zhang, Sixing Chen, Sandra Lapinska, Alexander O. Flynn-Carroll, Bogdan Pasaniuc, Douglas J. Epstein, and Iain Mathieson

Supplemental Note

Power Analysis

To evaluate the ability of our study to detect genetic associations given the relatively low prevalence of Ménière's disease (MD) and case-control imbalance, we performed a statistical power analysis. Power was assessed as a function of minor allele frequency (MAF) and effect size (odds ratio, OR) at genome-wide significance ($P < 5\times10^{-8}$), using a standard non-centrality parameter framework for case-control association tests[1,2] (**Figure S1**). Specifically, for each combination of MAF and OR, we derived the expected allele frequency difference between cases and controls under a multiplicative model and computed the corresponding non-centrality parameter, accounting for the number of cases and controls in our study. Power was then obtained from the non-central chi-square distribution with one degree of freedom.

We observed that statistical power is primarily driven by the number of cases, given the extreme imbalance between cases and controls, with an effective sample size of 35,713. Under this framework, our study has high power (>80%) to detect common variants (MAF ≥ 0.05 and 0.1) with modest effect sizes (OR = 1.356 and 1.248), whereas substantially larger effects (OR = 1.929) are required for low-frequency variants (MAF = 0.01). These results indicate that our study is well-powered to identify common risk variants of modest effect but has limited sensitivity for detecting low-frequency variants unless their effects are large. This provides important context for interpreting both detected associations and the absence of signals at lower allele frequencies.

These power estimates should be interpreted in the context of the inherent challenges of studying a low-prevalence disorder such as Ménière's disease. With a population prevalence of approximately 0.2-0.5%, achieving a large case cohort requires aggregating data across multiple large-scale biobanks, as performed here. Our study, with 8,969 cases, represents one of the largest genetic studies of MD to date, and future progress will require additional MD-enriched cohorts.

References

1. Skol, A.D., Scott, L.J., Abecasis, G.R., and Boehnke, M. (2006). Joint analysis is more efficient than replication-based analysis for two-stage genome-wide association studies. Nat. Genet. *38*, 209–213. https://doi.org/10.1038/ng1706.

2. Purcell, S., Cherny, S.S., and Sham, P.C. (2003). Genetic Power Calculator: design of linkage and association genetic mapping studies of complex traits. Bioinformatics *19*, 149–150. https://doi.org/10.1093/bioinformatics/19.1.149.

Supplemental Figures

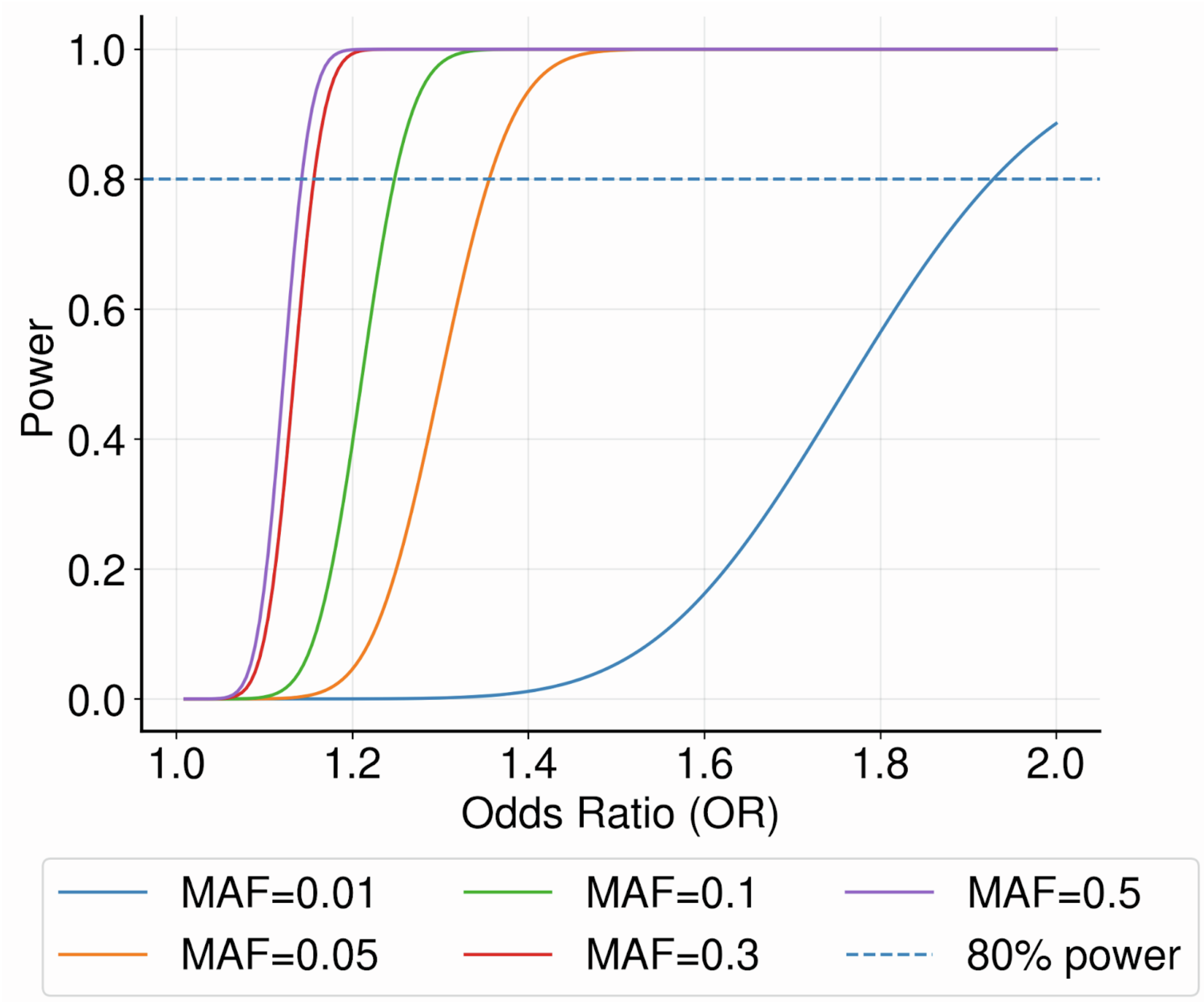

Figure S1. Power analysis for case–control GWAS.
Statistical power to detect genetic associations at genome-wide significance ($P < 5\times10^{-8}$) is shown as a function of odds ratio (OR) for variants with minor allele frequencies (MAF) of 0.01, 0.05, 0.1, 0.3, and 0.5 and 8,969 cases and 1,962,542 controls. The horizontal dashed line denotes 80% power.

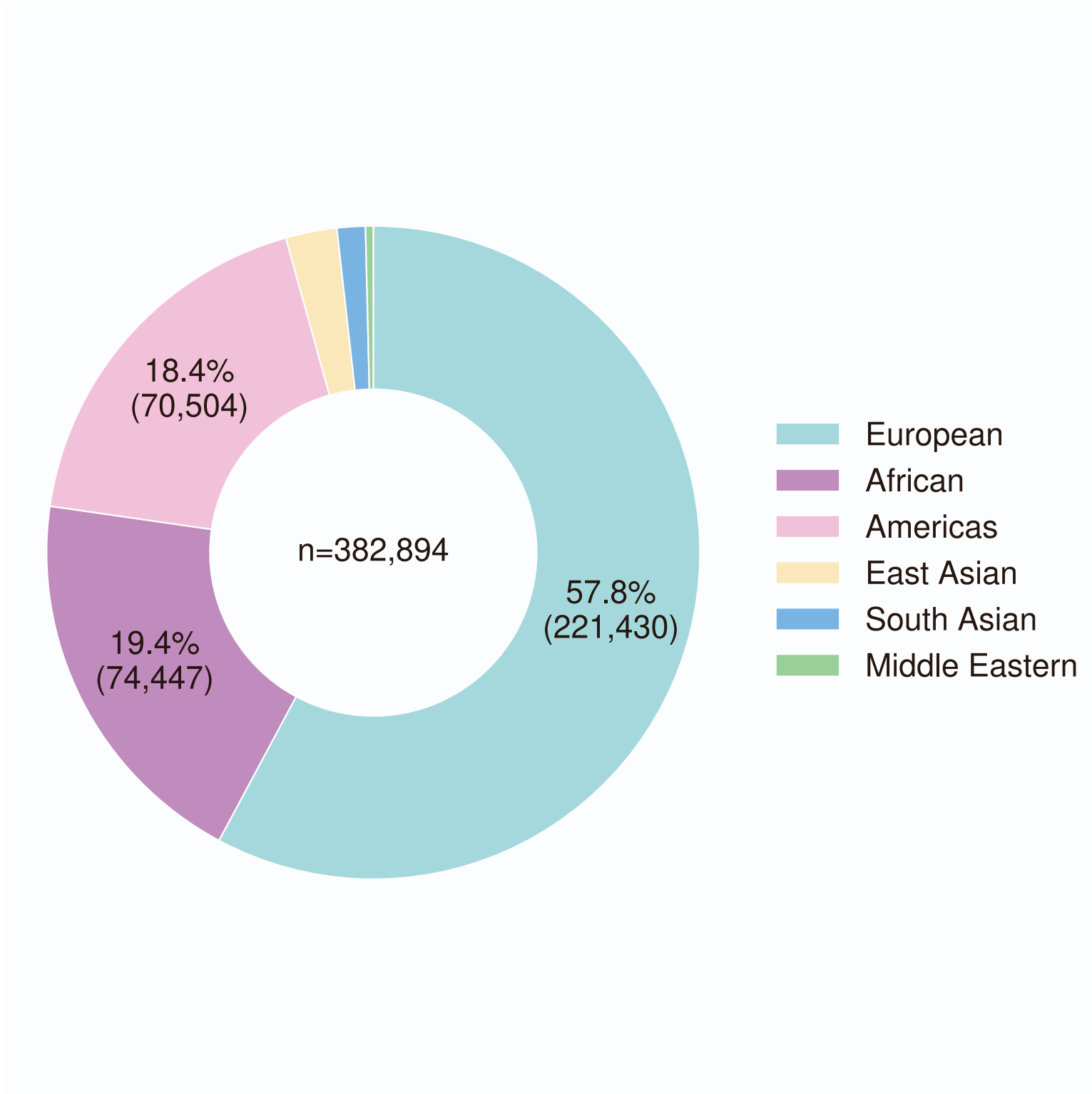

Figure S2. Genetically inferred ancestry distribution of all individuals in the All of Us version 8.

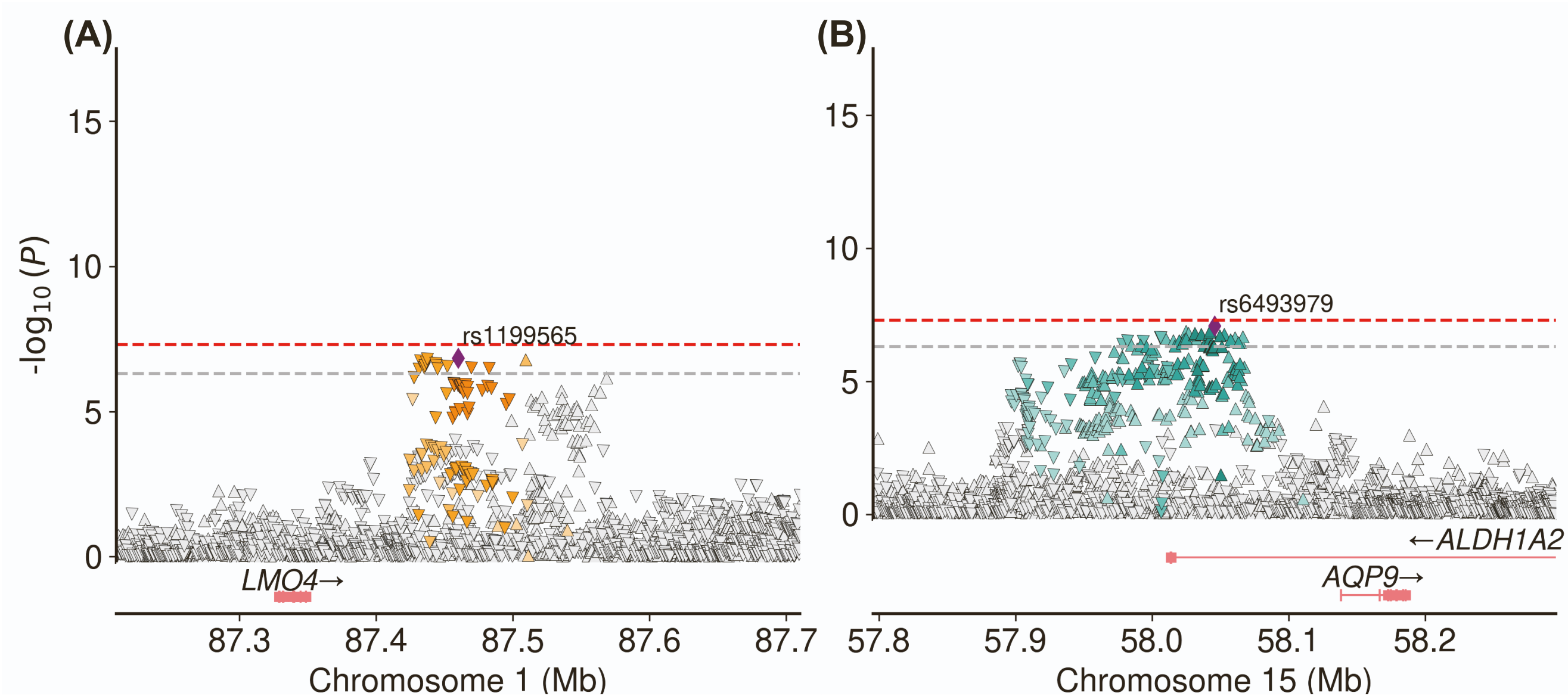

Figure S3. LocusZoom plot of suggestive loci for Ménière's disease.
Negative $\log_{10}$ P-values from the meta-analysis plotted against genomic positions for each index SNP region. The window size of the plots is 500kb. Genes of interest are colored in red. Red dotted line denotes genome-wide significance ($P<5\times10^{-8}$). Grey dotted line denotes suggestive significance ($5\times10^{-8}<P<5\times10^{-7}$).

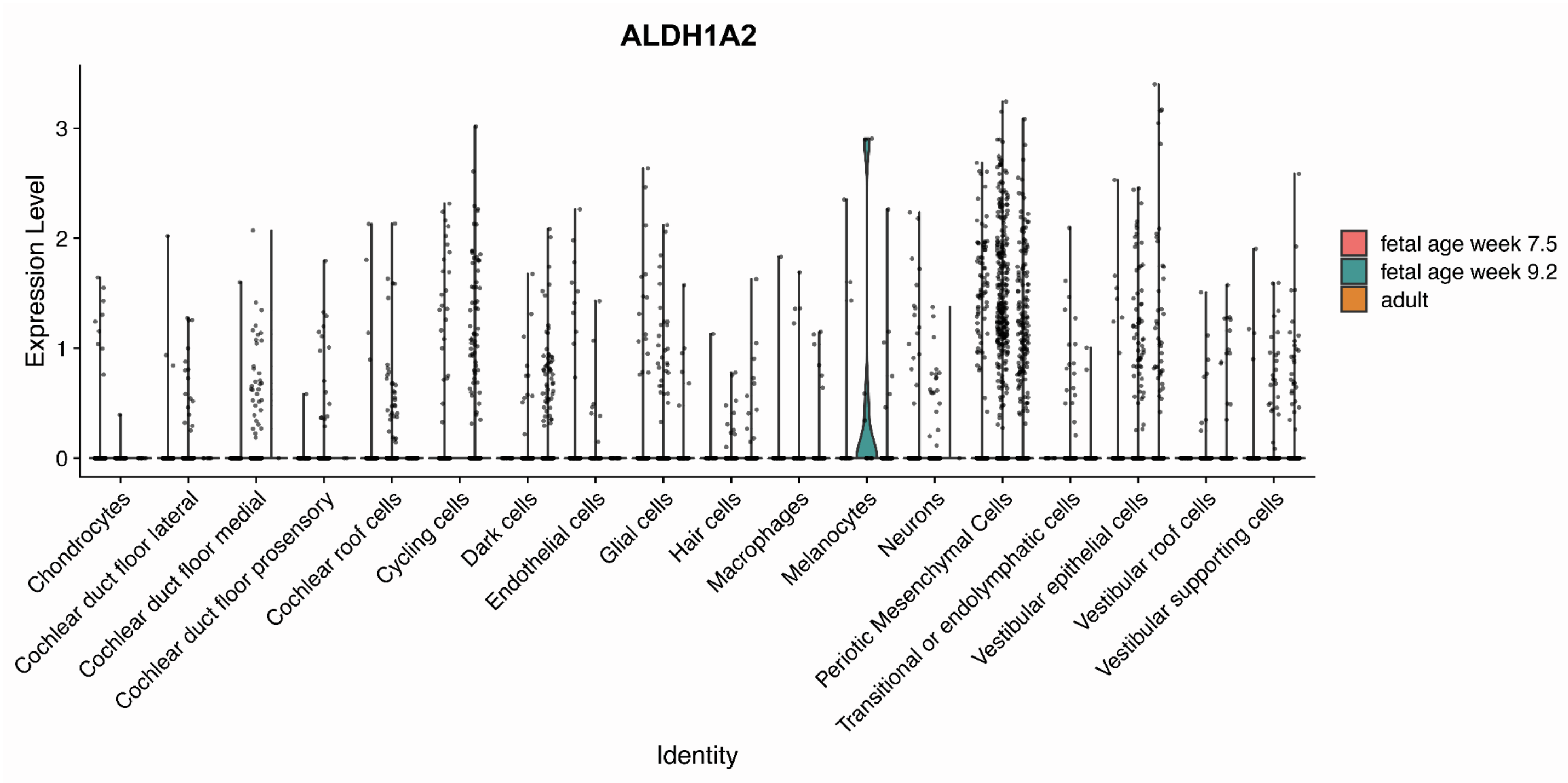

Figure S4. Violin plot of single-cell expression in ALDH1A2 from human inner ear scRNAseq across developmental stages.

A violin plot shows the expression of ALDH1A2 across diverse human inner ear cell types at three developmental stages (fetal week 7.5, fetal week 9.2, and adult). Data were obtained from the human inner ear atlas[60] *via the gEAR Dashboard*[61].

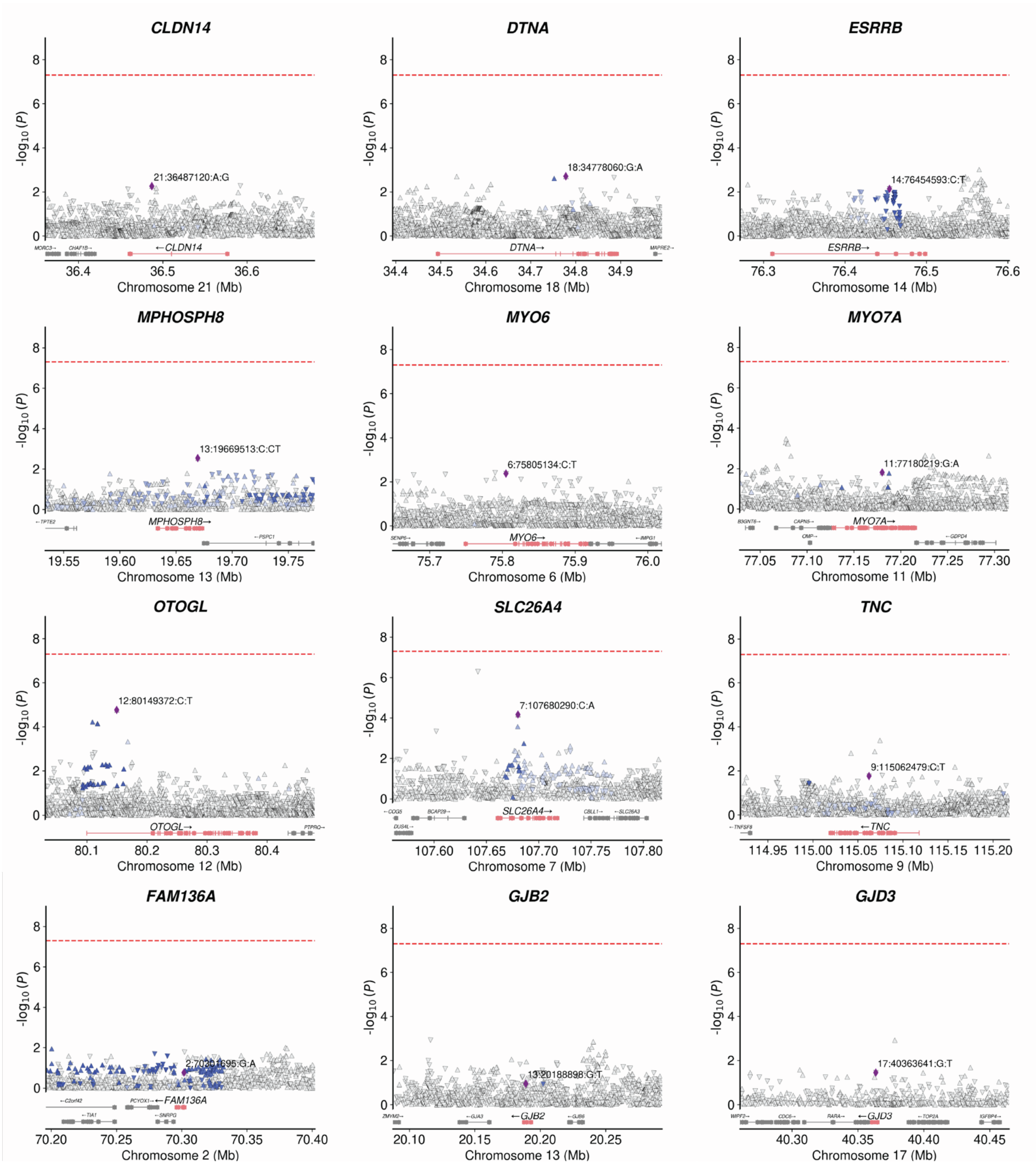
CLDN14
21:36487120:A:G
Chromosome 21 (Mb)
DTNA
18:34778060:G:A
Chromosome 18 (Mb)
ESRRB
14:76454593:C:T
Chromosome 14 (Mb)
MPHOSPH8
13:19669513:C:CT
Chromosome 13 (Mb)
MYO6
6:75805134:C:T
Chromosome 6 (Mb)
MYO7A
11:77180219:G:A
Chromosome 11 (Mb)
OTOGL
12:80149372:C:T
Chromosome 12 (Mb)
SLC26A4
7:107680290:C:A
Chromosome 7 (Mb)
TNC
9:115062479:C:T
Chromosome 9 (Mb)
FAM136A
2:70301695:G:A
Chromosome 2 (Mb)
GJB2
13:20188898:G:T
Chromosome 13 (Mb)
GJD3
17:40363641:G:T
Chromosome 17 (Mb)
-log10 (P)

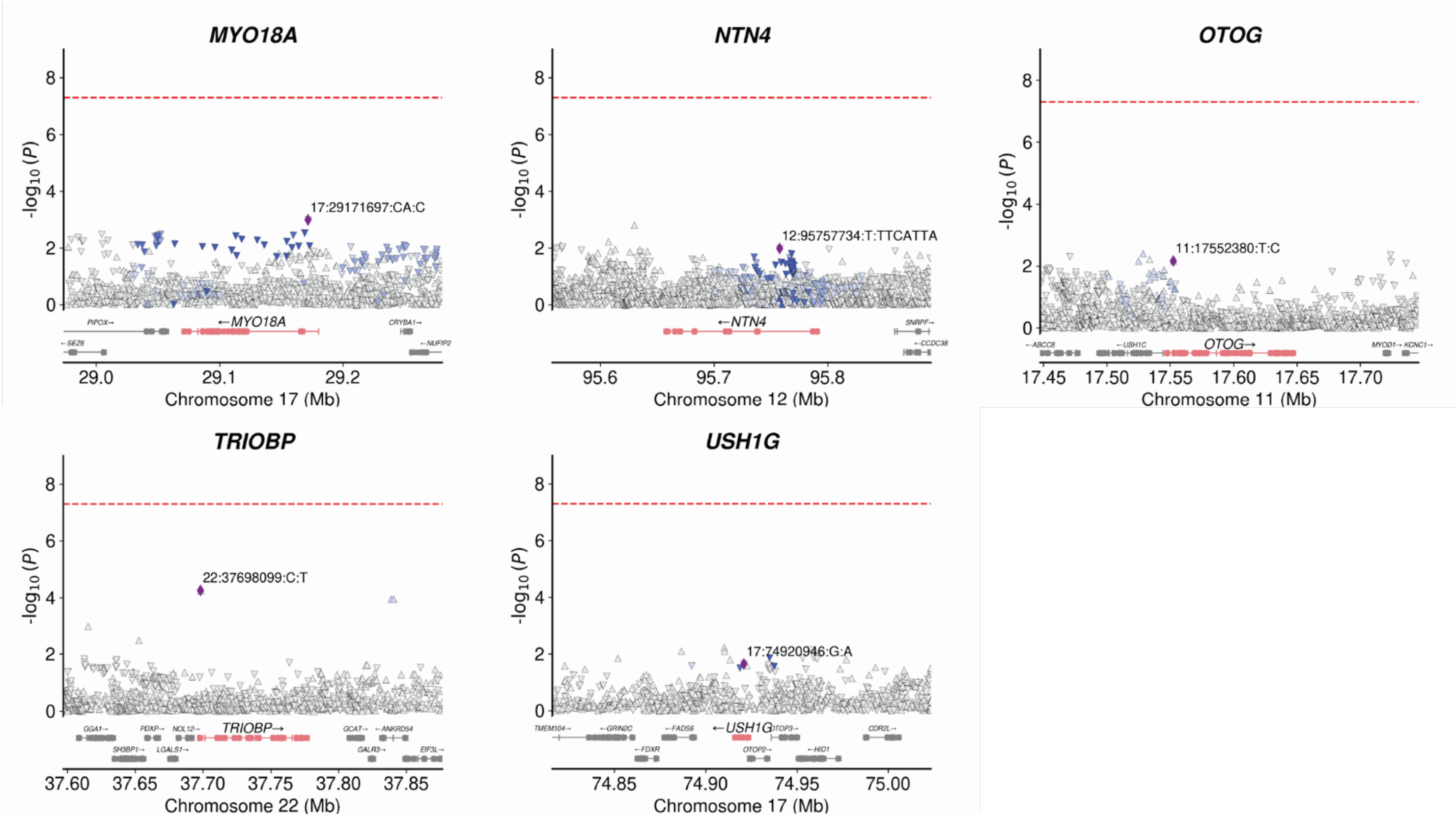

Figure S5: LocusZoom plots for regions around previously reported genes.

-log10 P-values from meta-analysis plotted against genomic positions for each previously reported gene[12-21]. Genes were identified via burden test or familial studies. The window size for the plots is ±100kb around the gene's start and end positions. Genes of interest were colored in red along the x-axis. Red dotted line denotes genome-wide significance (P<5×10-8). The variant with the lowest P-value and non-zero MAF in the 1000 Genomes EUR within each gene body was annotated.
